# Formalizing the use case model: A model-based approach

**DOI:** 10.1371/journal.pone.0231534

**Published:** 2020-04-20

**Authors:** Qamar uz Zaman, Aamer Nadeem, Muddassar Azam Sindhu

**Affiliations:** 1 Department of Computer Science, Capital University of Science and Technology (CUST), Islamabad, Pakistan; 2 Department of Computer Science, Quaid-i-Azam University, Islamabad, Pakistan; Consejo Superior de Investigaciones Cientificas (CSIC), SPAIN

## Abstract

In general, requirements expressed in natural language are the first step in the software development process and are documented in the form of use cases. These requirements can be specified formally using some precise mathematical notation (e.g. Linear Temporal Logic (LTL), Computational Tree Logic (CTL) etc.) or using some modeling formalism (e.g. a Kripke structure). The rigor involved in writing formal requirements requires extra time and effort, which is not feasible in several software development scenarios. A number of existing approaches are able to transform informal software requirements to formal specifications. However, most of these approaches require additional skills like understanding of specification languages additional artifacts, or services of domain expert(s). Consequently, an automated approach is required to reduce the overhead of effort for converting informal requirements to formal specifications. This work introduces an approach that takes a use case model as input in the proposed template and produces a Kripke structure and LTL specifications as output. The proposed approach also considers the common use case relationships (i.e., *include* and *extend*). The generated Kripke structure model of the software allows analysis of software behavior early at the requirements specification stage which otherwise would not be possible before the design stage of the software development process. The generated LTL formal specifications can be used against a formal model like a Kripke structure generated during the software development process for verification purpose. We demonstrate the working of the proposed approach by a SIM vending machine example, where the use cases of this system are inputs in the proposed template and the corresponding Kripke structure and LTL formal specifications are produced as final output. Additionally, we use the NuSMV tool to verify the generated LTL specifications against the Kripke structure model of the software, which reports no counterexamples thus validating the proposed approach.

## Introduction

Precise, consistent and verifiable software requirements are more useful for software verification and validation activities than ambiguous, inconsistent and unverifiable software requirements written in a natural language. These features of software requirements are mainly dependent on the selected requirements specification approach [1]. Informal software specification is quite flexible due to the use of natural language. However, natural language requirements are prone to errors and ambiguities. Consequently, the much needed characteristics of a software specification, i.e., clarity and correctness can get compromised. Moreover, it reduces the chances for providing regular and predictable support services which are usually required after the deployment of a software [2]. In contrast, when these requirements are formally specified, they ensure a higher degree of consistency, reliability and extendibility. These specifications, due to their well defined syntax and semantics are unambiguous. However, formal specifications are highly demanding in terms of time and effort [3]. The required additional time and cost, may not be feasible in all development scenarios. This creates space for the development of an approach that can transform informal software requirements into formal software requirements.

In the literature, a number of approaches can be found that transform informal software requirements to formal requirements. For example, approaches proposed by Somé et al. [4], Kalnins et al. [5] and Scandurra et al. [6]. These approaches require software requirements written in natural language and transform these to corresponding requirements in a formal language. Though these approaches perform well but they have different types of limitations, for example some of these depend upon domain specific ontology [7], others require expertise in supporting skills like formation of domain diagram, activity diagram, interaction diagram or class diagram [8]. In addition to these limitations, some of the approaches either use Restricted Use Case Modeling (RUCM) [9], or Use case Specification Language (USL) [10] that requires understanding and usage of pre-defined syntax rules. Besides, these approaches lack the capability to handle the use case relationships, i.e., *include* and *extend* relationships which are useful for re-usability. Moreover, approaches proposed by Somé et al. [4], Kalnins et al. [5], Scandurra et al. [6] and Yue et al. [9] and Chu et al. [10] perform transformation at model level. A major limitation of model level transformation is that it is not a general purpose transformation and works only for some selected configurations. This limitation was addressed by proposing a meta-model based transformation mechanism. The distinctive characteristics of this setup lie in its capability to handle all possible features of the source model [11].

Realizing the importance of transformation and effects of overheads involved in the existing transformation approaches, this work proposes an approach that transforms use case descriptions into corresponding Kripke structure and LTL formal specifications. This approach requires to document the use case description(s) in the proposed template in a natural language. This approach also handles the commonly used use case relationships, i.e., *include* and *extend* relationship. It performs the transformation at meta-model level. Both generated LTL formal specifications and Kripke structure can be used as input to model checkers like NuSMV [12], SPIN [13] and SAL [14]. However, model checking is not a direct subject of the current study; instead, the focus is on formalization of use case description. The major contributions of this paper are: meta-models for use case and Kripke structure and an approach to transform use case description into a Kripke structure at meta-model level and use case to LTL specifications directly.

The proposed meta-models for use case and Kripke structure as well as the proposed approach are discussed in the proposed approach section, use case model and the generated Kripke structure along with the generated LTL formal specifications for SIM vending machine example are presented in the example section. Related work section discusses the existing state-of-the-art approaches in this context. Finally, the conclusion section concludes the work.

## Proposed approach

**Fig 1:**
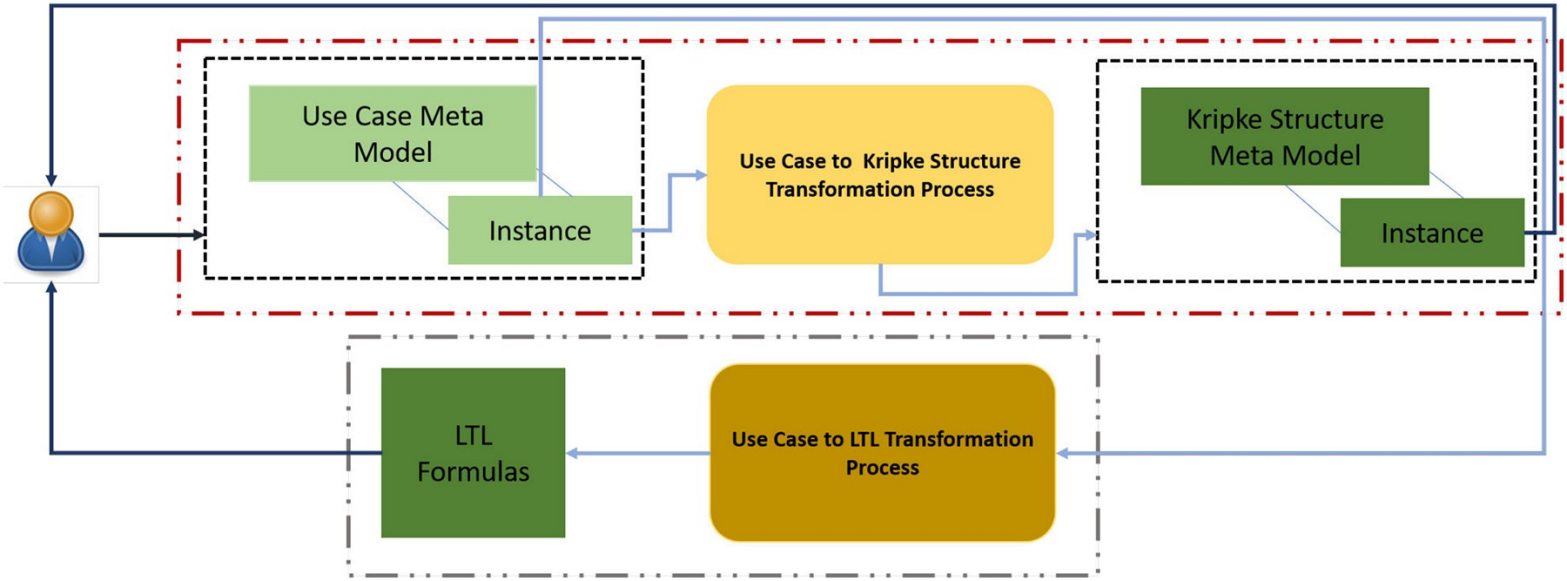
Proposed approach schematic diagram.

This paper presents an approach to transform use case description(s) to a Kripke structure and LTL formal specifications. The use case description(s) is(are) required to be specified in the proposed use case template using a natural language. The proposed template requires to specify a use case description using a set of keywords along with the distinct listing of software input and output symbols. These input and output symbols are identified at the requirements elicitation stage. To make the proposed approach suitable for model-driven object-oriented paradigm, meta-models for use case description and Kripke structure have been defined. Along with these meta-models, transformation rules that transform a use case description to a Kripke structure and LTL formal specifications are also defined. A user can use either of these as an input to the model checkers like NuSMV, SPIN and SAL to verify the software behavior at an early stage of software development process which otherwise would not be possible before the design phase of the software development process. Fig 1 shows a schematic diagram of the proposed approach.

The user populates an instance of the use case model. This populated use case instance is processed by *Use Case to Kripke Structure Transformation Process* and a Kripke structure is generated as an output. *Use Case to LTL Transformation Process* generates LTL formal specifications(LTL formulas) from the use case description provided as an input. The proposed meta-models for use case description and Kripke structure along with *Use Case to Kripke Structure Transformation Process* and *Use Case to LTL Transformation Process* are discussed in the following sub-sections.

### Use case meta-model

Generally, a use case template is required for writing a use case description. There are a number of available use case templates. Examples of popular use case templates include the templates proposed by Cockburn [15], Ivar Jacobson [2], RUP [16], Duran [17], Leite [18] etc. These available use case templates contain some common features like use case name, actor name, success scenario and alternate scenario. But to the best of our knowledge, UML does not recommend any template as a standard template. This allows to propose a new use case template, if required.

As discussed earlier, there are multiple available use case templates, but none of these lists input and output symbols of the software explicitly. Moreover, available templates list alternate scenario(s) in a separate section. This makes it difficult to proceed for the transformation process as the transformation process must repeatedly scan the use case description back and forth to track the possible flow of the use case. To overcome these difficulties and to make the transformation process simple, we propose a use case template that lists the input and output symbols explicitly. Furthermore, it enlists the alternate scenario(s) along with the normal scenario.

Furthermore, the use case relationships are used to reuse a use case to make the system more operable. This makes a use case more flexible to write in a more customized format. Zaman et al. [19] proposed a use case template that is closer to our requirements of writing a use description. However, this template does not handle use case relationships, i.e., *include* and *extend*. In addition, their proposed template requires the *lengthofBitVector*, *BitVector* and binary values for output symbols. These requirements make this template difficult to use by a common user. The proposed template in this work does not require the user to calculate *lengthofBitVecor*, *BitVector* and corresponding binary values for output symbols. In addition, this template also handles use case relationships. An *include* relationship allows to include another use case whereas, the *extend* relationship extends a use case functionality on some specified interaction. As an example, consider the case of a software that allows a user to choose a payment option by selecting to pay using a credit card or to pay by cash on delivery. This software also requires the user to re-login when finalizing the payment option. Use case relationships facilitate to specify this scenario.*Pay by card* or *pay by cash on delivery* use cases can be extended and the requirement to re-login can be documented by including the *login* use case.

**Fig 2:**
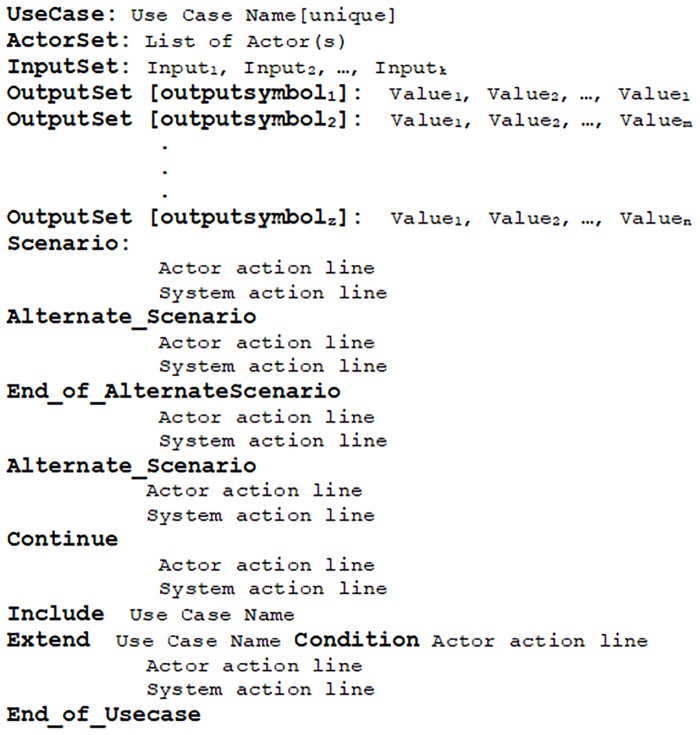
Proposed use case template.

A specimen of the proposed use case template is given in Fig 2.

The proposed template consists of a set of keywords including *UseCase*:, *ActorSet*:, *InputSet*:, *OutputSet*:, *Scenario*, *Alternate_Scenario*:, *End_of_AlternateScenario*, *Continue*, *Include*, *Extend*, *Condition* and *End_of_Usecase*.

The use case name is required to be unique in a software and is recorded with the keyword *UseCase*:. Software’s actor(s) is(are) labeled with the keyword *ActorSet*:. This template allows to record input and output symbols explicitly. It enlists the possible input with a keyword *InputSet*:. For example, a user’s credentials may be valid or invalid. In this case, the *InputSet*: will have *valid_credentials* and *invalid_credentials*. Each use case is required to have an *InputSet*:. The output set is denoted by *OutputSet*: keyword and contains the possible output values for this output symbol, e.g., a system can display a successful login message or invalid login attempt, depending on the provided credentials to the software by a user. In this case, the *OutputSet*: with a *label* login message contains *successful_login_message* and *invalid_login_attempt*. Other possible *OutputSet*: may have output symbols like *file_uploaded_successfully* and *invalid_file_upload_attempt* with a *label* file notification message. The proposed approach is flexible and does not place any limitation on the number of *OutputSet*: and output symbols. It dynamically fulfills the contextual requirements.

An actor’s interaction with a software is listed under the keyword *Scenario* and a possible alternate scenario is with the keyword *Alternate_Scenario*. An alternate scenario can be concluded in two ways either with *Continue* or *End_of_AlternateScenario* keyword. An alternate scenario ended with *End_of_AlternateScenario* keyword represents an interaction with the software which results in halting the execution and switching to the position from where the alternate scenario started whereas, the *Continue* keyword marks the end of alternate scenario where a software continues to repeat its operation unless a valid input is provided. The keyword *End_of_Usecase* is used to mark the end of a use case. The keywords *Include* and *Extend* are used to specify the use case relationships namely include and extend respectively and require a valid use case name. There is another keyword *Condition* that specifies the user interaction with the software when an extending use case extends the specified use case.

**Fig 3:**
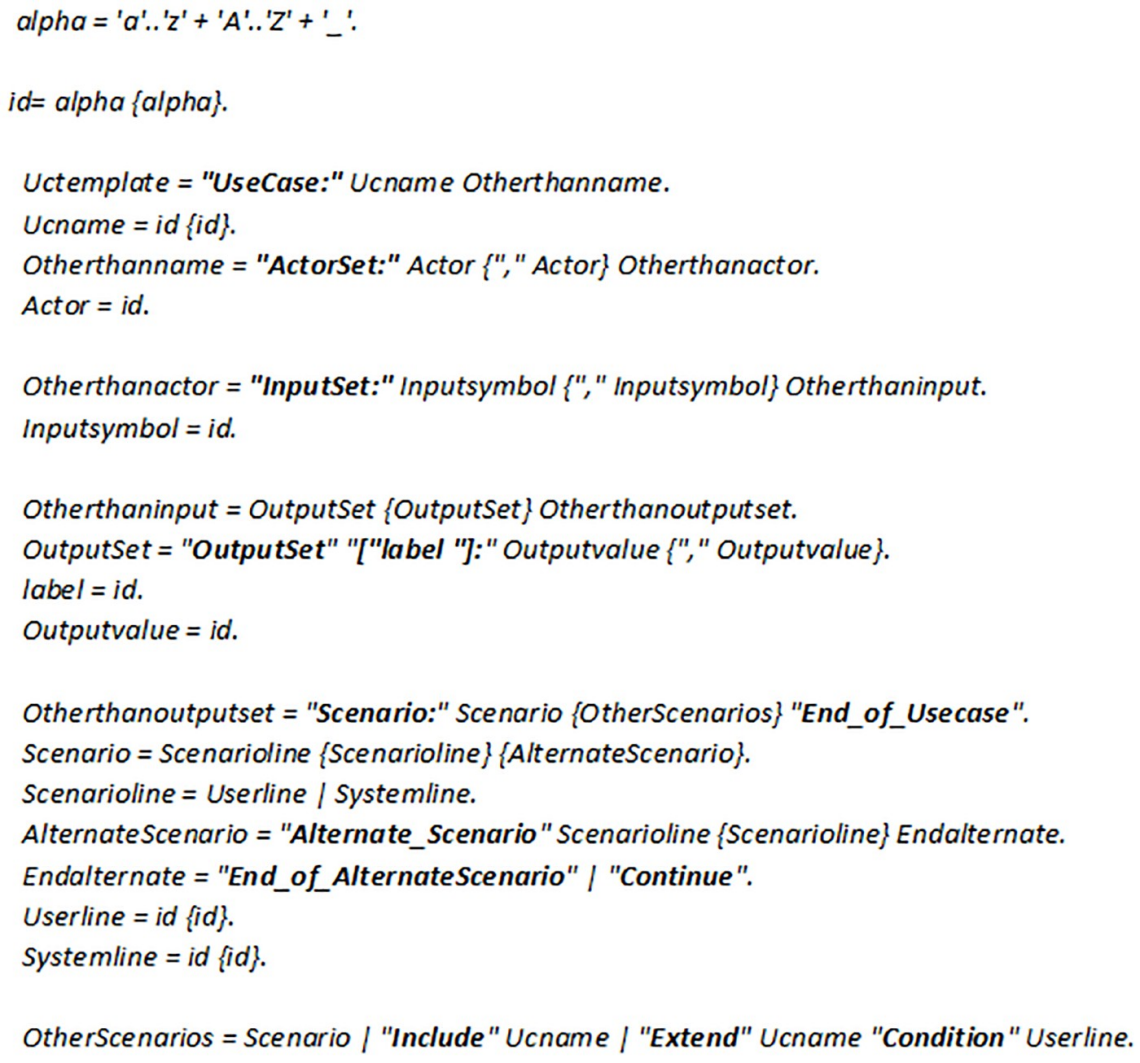
Context free grammar for use case template.

A context free grammar has been defined for the proposed use case template using Extended Backus-Naur Form (EBNF) notation Fig 3. To ensure syntactical correctness of the input use case, a parser has been developed. Fig lists the context free grammar. An *id* is defined as a string of alphabets and _. This id can be used to define *Ucname*, *inputsymbol*, output’s *label*, *Outputvalue*, *Userline* and *Systemline*. A *Ucname* is used with *UseCase*: to specify the name of a use case. In addition, it is also used to specify the name of use case being included or extended. The use case being included is specified with *Include*, whereas the use case being extended is specified with *Extend* along with *Condition*. The actors of a use case are listed with *ActorSet*: keyword. The input symbols of a use case can be specified by *InputSet*:. The output symbols are listed with *OutputSet* along with its possible values. The *Scenario*: lists the user action line(s) and system action line(s). The scenario is ended with *End_of_Usecase*. All the possible alternate scenarios are listed with *Alternate_Scenario*. It lists the alternate user line(s) and system line(s). An alternate scenario can be ended either by *End_of_AlternateScenario* or *Continue*.

A use case containing *Include* or *Extend* is flattened by *Use Case Flattener* process and it is listed as Rule 1. This process accepts a use case description, read its scenario line by line for the occurrence of *Include* or *Extend*. If any of these is found it calls *Use Case Includer* or *Use Case Extender* rules accordingly.

**Rule 1** Use Case Flattener

**Require**: *UC* as a use case description in the proposed template

**Ensure**: *UC*_*flattened*_ as a use case description in the proposed template

1: Define *UC*_*temp*_, *UC*_*flattened*_.*ActorSet* ← *UC*.*ActorSet*, *UC*_*flattened*_.*InputSet* ← *UC*.*InputSet*, *UC*_*flattened*_.*OutputSet* ← *UC*.*OutputSet*

2: **for** *ℓ* in *UC*.*Scenario* **do**

3:  **if** *ℓ* contains *Include* **then**

4:   *UC*_*temp*_ ← *Ucname*

5:   *UC*_*flattened*_ ← IncludeUseCase(*UC*_*flattened*_,*UC*_*temp*_)

6:  **else if** *ℓ* contains *Extend* **then**

7:   *UC*_*temp*_ ← *Ucname*

8:   *UC*_*flattened*_ ← ExtendUseCase(*UC*_*flattened*_,*UC*_*temp*_,Userline)

9:  **else**

10:   *UC*_*flattened*_.*Scenario* ← *UC*_*flattened*_.*Scenario* + *ℓ*

11:  **end if**

12: **end for**

**Rule 2** Use Case Includer

**Require**: *UC*_*flattened*_, *UC*_*included*_ as use case descriptions in the proposed template

**Ensure**: *UC*_*flattened*_ as a use case description in the proposed template

1: *UC*_*flattened*_.*ActorSet* ← *UC*_*flattened*_.*ActorSet* ∪ *UC*_*included*_.*ActorSet*

2: *UC*_*flattened*_.*InputSet* ← *UC*_*flattened*_.*InputSet* ∪ *UC*_*included*_.*InputSet*

3: *UC*_*flattened*_.*OutputSet* ← *UCflattened*.*OutputSet* ∪ *UC*_*included*_.*OutputSet*

4: *UC*_*flattened*_.*Scenario* ← *UC*_*flattened*_.*Scenario* + *UC*_*included*_.*Scenario*

**Rule 3** Use Case Extender

**Require**: *UC*_*flattened*_, *UC*_*extended*_ as use case descriptions in the proposed template, Userline as scenario line

**Ensure**: *UC*_*flattened*_ as a use case description in the proposed template

1: *UC*_*flattened*_.*ActorSet* ← *UC*_*flattened*_.*ActorSet* ∪ *UC*_*extended*_.*ActorSet*

2: *UC*_*flattened*_.*InputSet* ← *UC*_*flattened*_.*InputSet* ∪ *UC*_*extended*_.*InputSet*

3: *UC*_*flattened*_.*OutputSet* ← *UCflattened*.*OutputSet* ∪ *UC*_*extended*_.*OutputSet*

4: *UC*_*flattened*_.*Scenario* ← *UC*_*flattened*_.*Scenario* + *Extension*_*Point* + *Userline*

5: *UC*_*flattened*_.*Scenario* ← *UC*_*flattened*_.*Scenario* + *UC*_*extended*_.*Scenario* + *End*_*Extension*_*Point*

Rule 2 takes *UC*_*flattened*_ and *UC*_*included*_ use case descriptions. It combines *ActorSet*, *InputSet* and *OutputSet* of both use case descriptions. Moreover, this process appends scenario lines of *UC*_*included*_ to the scenario lines of *UC*_*flattened*_.

**Fig 4:**
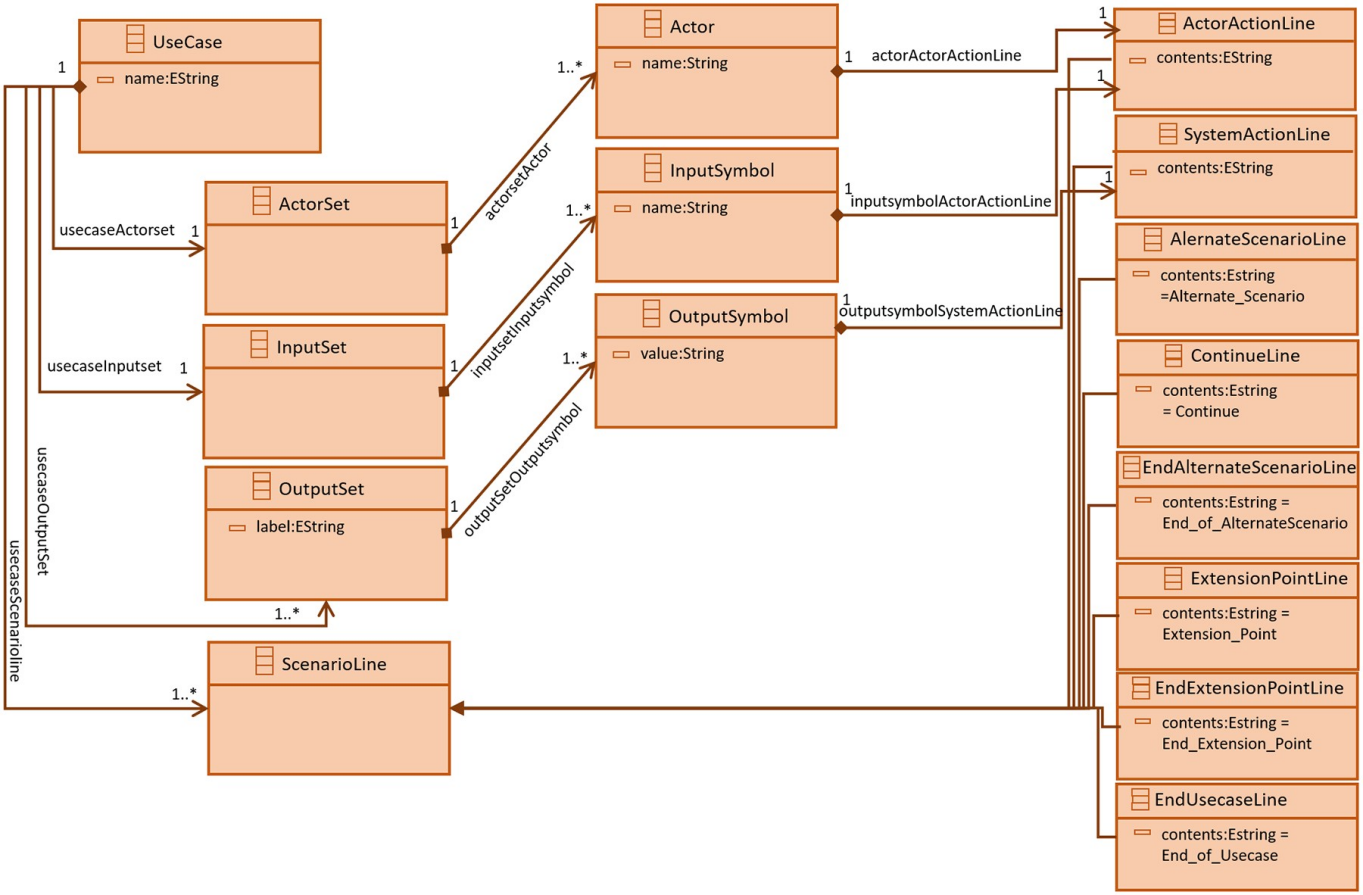
Use case meta-model.

Rule 3 accepts *UC*_*flattened*_ and *UC*_*extended*_ use case descriptions. It combines *ActorSet*, *InputSet* and *OutputSet* of both use case descriptions. This process then append *Extension_Point*, *Userline* and scenario lines of *UC*_*extended*_ use case description to the the *UC*_*flattened*_ scenario lines. This process ends by appending *End_Extension_Point* to the *UC*_*flattened*_ scenario. A meta-model for this flattened use case is defined and it is shown in Fig 4.

*UseCase* is the main element that includes *ActorSet*, *InputSet*, *OutputSet* and the *ScenarioLine*. *UseCase* element has a data member *name* to store use case name. *ActorSet* includes *Actor* element(s) with a data element *name* to store the value of an actor. The true strength of meta-model can be used if it carries its relationships with multiple objects of the same structure. For this matter mutual cardinality of objects is taken into account. There is one to one cardinality between *UseCase* and *ActorSet*, whereas the cardinality between *ActorSet* and *Actor* elements is one to many. *UseCase* can only have one *InputSet* and there can be more than one *InputSymbol* elements in an *InputSet*. There can be multiple *OutputSet* in a *UseCase* to contain the possible output symbols with their respective values. *OutputSymbol* element in an *OutputSet* is *value*. *ScenarioLine* element in a *UseCase* element is used to represent use case scenario lines. A *UseCase* element can have multiple *ScenarioLine* elements. *ScenarioLine* element has its specialized forms including *ActorActionLine*, *SystemActionLine*, *AlternateScenarioLine*, *EndUseCaseLine*, *EndAlternateScenarioLine*, *ContinueLine*, *ExtensionPointLine* and *EndExtensionPointLine*. *ActorActionLine* element represents a use case scenario line where an actor’s interaction with the system and *SystemActionLine* element is used to represent a software response to an actor. *AlternateScenarioLine* element lists the start of an alternate scenario and *EndAlternateScenarioLine* to mark the end of alternate scenario. The end of an alternate scenario can also be marked by a *ContinueLine* element. *ExtensionPointLine* element is used to mark the scenario lines of a use case being extended and *EndExtensionLine* is used to mark the end of the scenario lines of the use case being extended. *EndUsecaseLine* is used to mark the end of use case.

The use case meta-model can be described as:

*UseCase*_*metamodel*_ = 〈 *name*_*i*_, *ActorSet*_*i*_, *InputSet*_*i*_, (*k* × *OutputSet*)_*i*_, (*j* × *ScenarioLine*)_*i*_ 〉. where *i* = 1, …, *n*, represents the *i*^*th*^ instance of use case meta-model. *name*_*i*_ is a use case model name. *ActorSet*_*i*_ = {*Actor*_1_, *Actor*_2_, …, *Actor*_*p*_} and *p* ∈ ***N***.

*InputSet*_*i*_ = {*InputSymbol*_1_, *InputSymbol*_2_, …, *InputSymbol*_*q*_} where *q* ∈ ***N*** and *InputSymbol*ś value is stored in name.

The variable *k* is a positive integer and it is used to represent the number of *OutputSet* in the *i*^*th*^ instance of use case meta-model and *OutputSet* = {*label*, *OutputSymbol*_1_, *OutputSymbol*_2_, …, *OutputSymbol*_*s*_} where *s* ∈ ***N*** and the *label* records the textual output e.g. login message. *OutputSymbol* = {*value*. The element *value* denotes the textual value of output symbol e.g. *successful_login_message* or *invalid_login_attempt*.

The variable *j* ∈ ***N*** denotes the number of scenario lines in the *i*^*th*^ instance of use case meta-model. The *ScenarioLine* = {*ActorActionLine*, *SystemActionLine*, *AlternateScenarioLine*, *EndAlternateScenarioLine* *ContinueLine*, *ExtensionPointLine*, *EndExtensionPointLine*, *EndUseCaseLine*}. The proposed meta-model is implemented in Eclipse Modeling Framework (EMF) [20].

### Kripke structure meta-model

A Kripke structure [21] is a formal notation and is a five-tuple 〈*Q*, Σ, *δ*, *q_0_*, λ〉 where

- *Q* is a finite set of states,
- Σ is a finite set of input symbols,
- *δ*:*Q*×Σ → *Q* is a transition function,
- *q_0_* ∈ *Q* is the initial state,
- λ: *Q*→2^*AP*^ is a labeling function

The *AP* are atomic propositions describing some property of a system over a particular state. An extension of a Kripke structure is proposed by Meinke et al. [22] to use it as a multi-bit Moore machine with states labeled by Boolean bitvector that makes it useful for test case generation of reactive systems by Learning-based Testing (LBT). The Kripke structure used for this purpose is defined as:

- *Q* is a finite set of states,
- Σ is a finite set of input symbols,
- *δ*:*Q*×Σ→ *Q* is a transition function.
- *q_0_*∈ *Q* is the initial state,
- λ: *Q* → $B^{k}$ is a labeling function and (*b_1_*,…,*b_k_*) ∈ $B^{k}$, a Boolean bitvector, is an indexing of a set *AP* of *k* atomic propositions.

**Fig 5:**
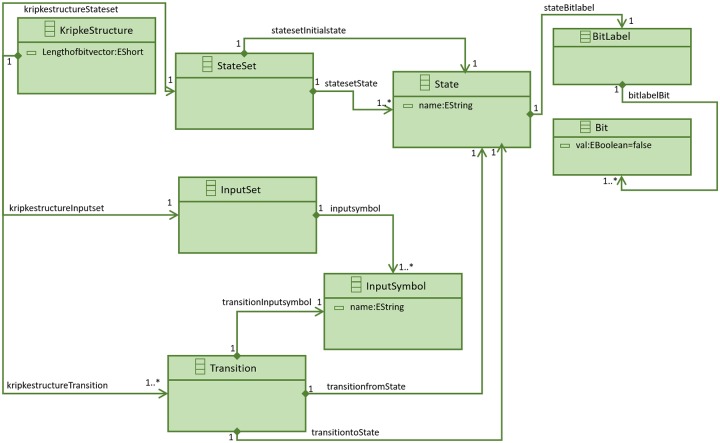
Kripke structure meta-model.

Model checkers like NuSMV [12], SPIN [13] and SAL [14] take a Kripke structure as input model for formal verification of software behavior. Fraser et al. [23] used a Kripke structure model to generate test cases for white box testing by exploiting structural properties of the software code using a Kripke structure model and in [24], [22] and [25], authors used specification based black box testing by learning Kripke structure models of the system under test. The model transformation process requires a meta-model of a Kripke structure. To the best of our knowledge, there is no existing meta-model definition for a Kripke structure. However, meta-model for a state machine exists in the literature [26]. In this study, we use the definition of Kripke structure as in [22]. A meta-model for the reformulated Kripke structure has been defined and is shown in Fig 5.

*KripkeStructure* element has a *StateSet* element which contains a start state and other states of a Kripke structure. A *State* element has a *name* element and a *BitLabel* to store the bitvector of the state. Its value is represented by *Bit* elements. Each *Bit* element can have a possible *true* or *false* value. A *KripkeStructure* element has an *InputSet* element that consists of multiple *InputSymbol* elements. The *InputSymbol* element has a *name*. A *KripkeStructure* element can have multiple *Transition* elements that represent the transitions of a Kripke structure. A *Transition* element is defined by a from and end state of *State* type and a transition symbol of type *InputSymbol*.

The Kripke structure meta-model can be represented as:

*KripkeStructure*_*metamodel*_ = 〈*StateSet*_*i*_, *InputSet*_*i*_, (*z*× *Transition*)_*i*_ 〉 where *i* = 1,…,*n* denotes the *i*^th^ instance of Kripke structure meta-model.

The element *StateSet*_*i*_ = {*q*_*initiali*_, *q*_1_, *q*_2_, …, *q*_*l*_} where *l* ∈ ***N***.

The initial state *q*_*initiali*_ = {*name* = *Initial_State* and *BitLabel*} where *BitLabel* = *Bit*_1_, …, *Bit*_*lengthofbitvector*_ and *Bit* = {*false*}.

The state *q* = {*name*, *BitLabel*} where *BitLabel* = *Bit_1_*, …, *Bit*_*lengthofbitvector*_ and *Bit* = {*true*, *false*}

The element *InputSet*_*i*_ = {*InputSymbol*_1_, *InputSymbol*_2_, …, *InputSymbol*_*m*_} where *m* ∈ ***N*** and *InputSymbol*= name.

The element *Transition* = {*q*_*fromstate*_, *q*_*tostate*_, *InputSymbol*}. A Kripke structure can have multiple transitions. The designed meta-model is implemented using EMF [20].

### Use case to Kripke structure transformation process

In model to model transformation, a model of a meta-model can be transformed to a model of another meta-model. This transformation can be automated if the transformation rules are expressed in some transformation language. Epsilon Transformation Language (ETL) is one such language [27]. It is is a hybrid model to model and rule based transformation language. It is built on top of the Epsilon model management platform that allows to perform multiple model management tasks including update in place, model to model and model to text transformation. ETL can transform many input models to many output models.

**Fig 6:**
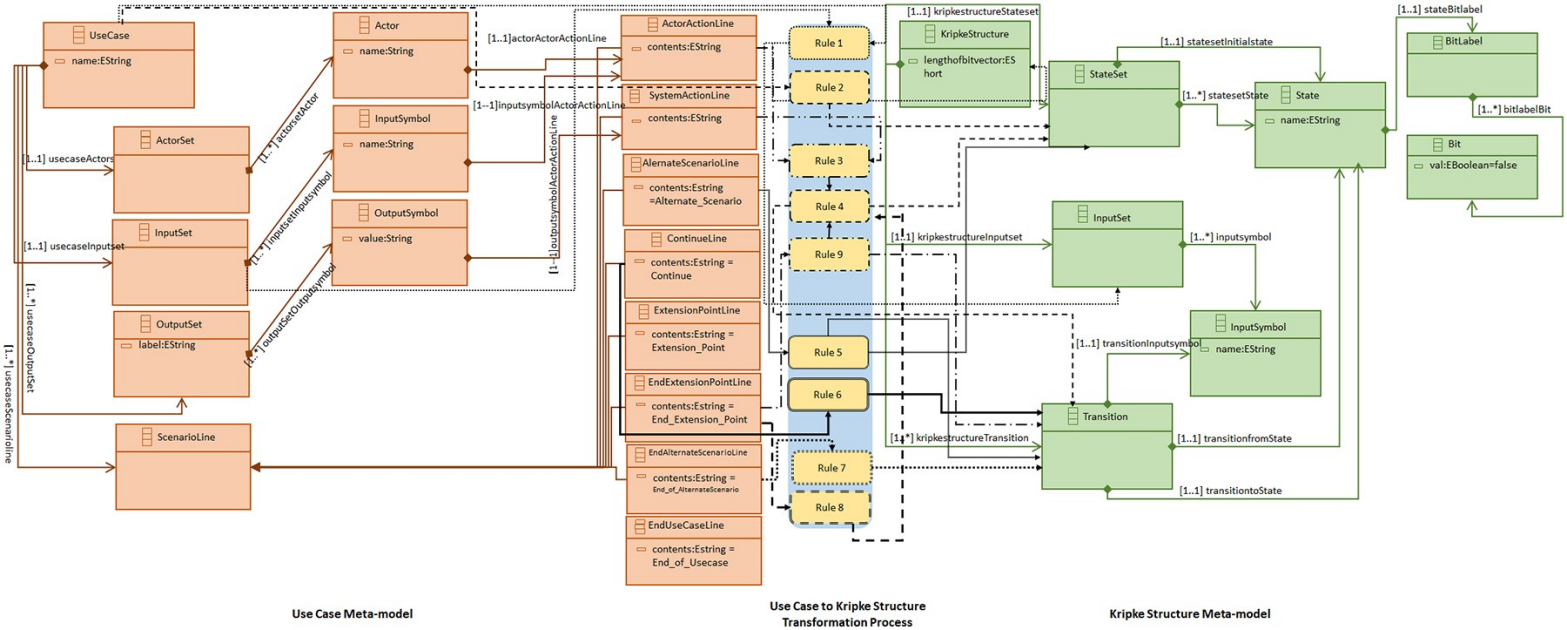
Use case to Kripke structure transformation.

Fig 6 shows the transformation process of a use case model to a Kripke structure model. A use case description *UC* is provided as an input model to this transformation process. The transformation process consists of nine rules to handle the provided use case description and to generate the resultant Kripke structure model *KS*. We will briefly discuss these rules in the following paragraphs. We abstracted some of the implementation details to make these rules more readable.

Rule 1 copies the *UC*.*InputSet* to *KS*.*InputSet* and also calculates the *bitvectorlength* value. In addition, it also generates random binary values for *OutputSymbol* in *OutputSet*.

**Rule 1** Calculate binary values, bitvectorlength and copy input symbols

1: *ucOPSet*_*Binary*_: new *UseCase*!*OutputSet*, *bitvectorlength* ← 0, *InputSet*_*temp*_: new *KripkeStructure*!*InputSet*

2: **for** *UC*.*OutputSet* **do**

3:  *count*_*output*_ ← *outputOutputSet*.*OutputSymbol*.*count*

4:  *bit*_*req*_ ← RequiredBits*count*_*output*_; *bitvectorlength* += *bit*_*req*_

5:  **for** *OutputSymbol* **do**

6:   *ucOPSet*_*Binary*_.*value* ← *OutputSymbol*.*value*

7:   *ucOPSet*_*Binary*_.*binaryvalue* ← random binary value

8:  **end for**

9: **end for**

10: **for** *UC*.*InputSet*.*InputSymbol* **do**

11:  *InputSet*_*temp*_.*InputSymbol*.*name* ← *UC*.*InputSet*.*InputSymbol*.*name*

12: **end for**

13: *KS*.*Inputset* = *InputSet*_*temp*_

14: *KS*.*bitvectorlength* ← *bitvectorlength*

Rule 2 defines *state*_*dead*_, *KS*.*State*.*InitialState* and *q*_*c*_*urrent* states. It also initializes the *KS*.*State*.*InitialState* and *state*_*dead*_ *BitLable*’s indices to *false*. In addition, it set *q*_*c*_*urrent* value to *KS*.*State*.*InitialState*.

**Rule 2** Define Initial and Dead states

1: *BitLabel*_*temp*_: new *KS*.*BitLable*, *state*_*dead*_, *q*_*current*_: new *KS*.*State*

2: **for** *BitLabel*_*temp*_ **do**

3:  *BitLabel*_*temp*_.*Bit*.*val* ← *false*

4: **end for**

5: *KS*.*State*.*InitialState*.*BitLabel* ← *BitLabel*_*temp*_

6: *state*_*dead*_ ← *BitLabel*_*temp*_

7: *q*_*current*_ ← *KS*.*State*.*InitialState*

Rule 3 reads a scenario line at a time and tracks the occurrence of actor, input and output symbols. On their occurrence it sets the value of flag bits *isActor*, *isInput* and *isOutput* accordingly. It also tracks the values of last read input symbol to *σ*_*temp*_ and output symbol to *output*_*temp*_.

**Rule 3** Scan a scenario line for the occurrence of Actor, Input and Output Symbols

1: **for** *UC*.*ScenarioLine* **do**

2:  **for** *UC*.*InputSet* **do**

3:   **if** *ℓ* contains *σ* **then**

4:    *isInput* ← *true*, *σ*_*temp*_ ← *InputSymbol*.*name*

5:   **end if**

6:  **end for**

7:  **for** *UC*.*ActorSet* **do**

8:   **if** *ℓ* contains *Actor* **then**

9:    *isActor* ← *true*

10:   **end if**

11:  **end for**

12:  **for** *UC*.*OutputSet* **do**

13:   **for** *OutputSymbol* **do**

14:    **if** *ℓ* contains *OutputSymbol* **then**

15:     *output*_*temp*_ ← *OutputSymbol*.*value*

16:    **end if**

17:   **end for**

18:  **end for**

19: **end for**‘

Rule 4 defines a new state *q*_*new*_ on the occurrence of a actor and input symbol in a scenario line. It defines *BitLabel*_*temp*_ with the value of *qcurrent*. The value of *BitLabel*_*temp*_ is updated by the *Bit Label Updater* process. It updates the corresponding indices for the *BitLabel*_*temp*_ with the corresponding binary value of *output*_*temp*_. The updated *output*_*temp*_ value is then assigned to *q*_*new*_.*BitLablel*. The newly created state *q*_*new*_ is then added to *KS.State*. A transition from *qcurrent* and *q*_*new*_ is defined and is labeled with the value of *σ*_*temp*_. This newly created transition is added to the *KS.Transition*. Transitions for the *UC*.*InputSet* other than *σ*_*temp*_ are also defined from *q*_*current*_ and *state*_*dead*_. These transitions are also added to *KS*.*Transition*. The value of *q*_*current*_ is updated with the value of *q*_*new*_.

**Rule 4** Define a new state and transitions

1: **if** *isInput* AND *isActor* **then**

2:  *isInput* ← *false*, *isActor* ← *false*

3:  Define *q*_*new*_, *BitLabel*_*temp*_ ← *q*_*current*_.*BitLabel*

4:  *BitLabel*_*temp*_ ← BitLabelUpdater (*output*_*temp*_, *ucOPSet*_*Binary*_, *BitLabel*_*temp*_)

5:  *q*_*new*_.*BitLabel* ← *BitLabel*_*temp*_

6:  *KS*.*State*.*add*(*q*_*new*_)

7:  **if** *isExtensionPoint* **then**

8:   *KS*.*Transition*.add(*q*_*beforeExtension*_, *q*_*new*_, *σ*_*temp*_)

9:  **else**

10:   *KS*.*Transition*.add(*q*_*current*_, *q*_*new*_, *σ*_*temp*_)

11:  **end if**

12:  **for** *UC*.*InputSet*—*σ*_*temp*_ **do**

13:   *KS*.*Transitio*.add(*q*_*current*_, *state*_*dead*_, *σ*)

14:  **end for**

15:  *q*_*current*_ ← *q*_*new*_

16: **end if**

Rule 5 describes the computation steps that are performed when a scenario line *ℓ* of type *UC*.*AlternateScenarioLine* is read. A temporary state *q*_*hold*_ is defined and the value of *q*_*current*_ is copied to it. Moreover, a new state *q*_*n*_ *ew* is created. The value of *q*_*current*_.*BitLabel* is copied to *BitLabel*_*temp*_. The value of *BitLabel*_*temp*_ is updated with the binary value of last output symbol read. The updated *BitLabel*_*temp*_ is assigned to *q*_*new*_.*BitLable*. This rule also defines a transition from the state *q*_*current*_ to the newly created state *q*_*nes*_ and is labeled with the value of *σ*_*temp*_. This transition is added to the *KS*.*Transition*. Transitions for the all input symbols other that *σ*_*temp*_ are defined from *q*_*current*_ and *dead*_*state*_ and are also added to *KS*.*Transition*.

**Rule 5** Process a use case line of type Alternate Scenario

1: **if** *ℓ*.typeOf(*UC*.*AlternateScenarioLine*) **then**

2:  *q*_*hold*_ ← *q*_*current*_

3:  Define *q*_*new*_, *BitLabel*_*temp*_ ← *q*_*current*_.BitLabel

4:  *BitLabel*_*temp*_ ← BitLabelUpdater (*output*_*temp*_, *ucOPSet*_*Binary*_, *BitLabel*_*temp*_)

5:  *q*_*new*_.BitLabel ← *BitLabel*_*temp*_

6:  *KS*.*State*.add(*q*_*new*_)

7:  *KS*.*Transition*.add(*q*_*current*_, *q*_*new*_, *σ*_*temp*_)

8:  **for** *UC*.*InputSet*—*σ*_*temp*_ **do**

9:   *KS*.*Transition*.add(*q*_*current*_, *state*_*dead*_, *σ*)

10:  **end for**

11:  *q*_*current*_ ← *q*_*new*_

12: **end if**

Rule 6 defines a new transition from *q*_*current*_ to *q*_*current*_ and is labeled with *σ*_*temp*_, when a scenario line of type *UC*.*ContinueLine* is read. The value of *q*_*current*_ is also updated with the value of *q*_*hold*_.

**Rule 6** Process a use case line of type Continue

1: **if** *ℓ*.typeOf(*UC*.*ContinueLine*) **then**

2:  *KS*.*Transition*.*add*(*q*_*current*_, *q*_*current*_, *σ*_*temp*_)

3:  *q*_*current*_ ← *q*_*hold*_

4: **end if**

Rule 7 defines a transition form *q*_*current*_ to *KS*.*State*.*InitialState* on reading a line of type *UC*.*EndAlternateScenarioLine* and this transition is labeled with value of *σ*_*temp*_. The value of *q*_*current*_ is updated with the value of *q*_*hold*_.

**Rule 7** Process a use case line of type End Alternate Scenario

1: **if** *ℓ*.typeOf(*UC*.*EndAlternateScenarioLine*) **then**

2:  *KS*.*Transition*.add(*q*_*current*_, *KS*.*States*.*InititalState*,*σ*_*temp*_)

3:  *q*_*current*_ ← *q*_*hold*_

4: **end if**

Rule 8 processes a scenario line of type *UC*.*ExtensionPointLine* that results in the definition of a new state *q*_*beforExtension*_. The calue of state *q*_*current*_ is copied to the state *q*_*beforExtension*_ and a flag *isExtensionPoint* is marked to *true*.

**Rule 8** Process a use case line of type Extension Point

1: **if** *ℓ*.typeOf(*UC*.*ExtensionPointLine*) **then**

2:  *isExtensionPoint* ← *true*

3:  *q*_*beforeExtenstion*_ ← *q*_*current*_

4: **end if**

Rule 9 processed a scenario line of type *UC*.*EndExtensionPointLine* and defines a new transition from *q*_*current*_ to *q*_*beforeExtenstion*_ is defined and it is labeled withe *σ*_*temp*_. The value of *q*_*current*_ is assigned with the value of *q*_*beforeExtension*_ and the flag bit *isExtentionPoint* set to *false*. The scenario line of type *UC*.*EndUsecaseLine* does not produce any impact on the transformation process.

**Rule 9** Process a use case line of type End Extension Point

1: **if** *ℓ*.typeOf(*UC*.*EndExtensionPointLine*) **then**

2:  *KS*.*Transition*.add(*q*_*current*_, *q*_*beforeExtension*_, *σ*_*temp*_)

3:  *q*_*current*_ ← *q*_*beforeExtenstion*_

4:  *isExtensionPoint* ← *false*

5: **end if**

The time complexity for *Use Case to Kripke Structure Transformation* process is calculated and it is ***O***(*n*(*ip*+*os*(*op*)+*ac*)), where *n* denotes the number of scenario lines, *ip* denotes the number of input symbols, *os* denotes the number of output set, *op* denotes the number output symbols in an *os* and *ac* denotes the number of actors in the use case description of the use case provided as an input to this process.

### Use case to LTL transformation process

The use case description, described in the proposed template, is also used to produce LTL formal specifications. LTL formal specifications are built using LTL formulas. LTL formulas are built from finite sets of atomic propositions, the logical operators and the temporal operators. The temporal operators include:

- Next operator, represented by the symbol ○ or ***X***
- Eventually operator, represented by the symbol ◇ or ***F***
- Globally operator, represented by the symbol □ or ***G***
- Until operator, represented by the symbol ***U***

Formal semantics of LTL operators can be described with the help of Kripke structure. Let ***K*** be a Kripke structure and a path *ρ* = 〈q_0_, *q*_1_,.:., *q*_*n*_〉 corresponding to a word *ω* = *σ*_0_, *σ*_1_, …, *σ*_*m*_ ∈ Σ^*ω*^ is a sequence such as ∀*i* ≥ 0: *q*_*i*+1_ = *δ*(*q*_*i*_, *σ*_*i*_) and *q*_0_ is the initial state. The set Paths(***K***, *q*_0_) denotes all paths in ***K***, where *q*_0_ is the initial state of ***K***. If we consider *ϕ* and *ψ* as two syntactically well formed LTL formulas then semantics of LTL operators over path *ρ* can be described as:

- ***X*** *ϕ*: ***K***, *ρ* ⊨ ***X*** *ϕ* ≡ ***K***, *ρ*^1^ ⊨ *ϕ*
- $F\phi :K,\rho ⊧F\phi \equiv \exists \;i\in N:K,\rho^{i}⊧\phi$
- $G\phi :K,\rho ⊧G\phi \equiv \forall \;i\in N:K,\rho^{i}⊧\phi$
- $\phi U\psi :K,\rho ⊧\phi U\psi \equiv \exists \;i,j\in N:K,\rho^{j}⊧\psi \wedge \forall 0\leq i<j,K,\rho^{i}⊧\phi$

**Fig 7:**
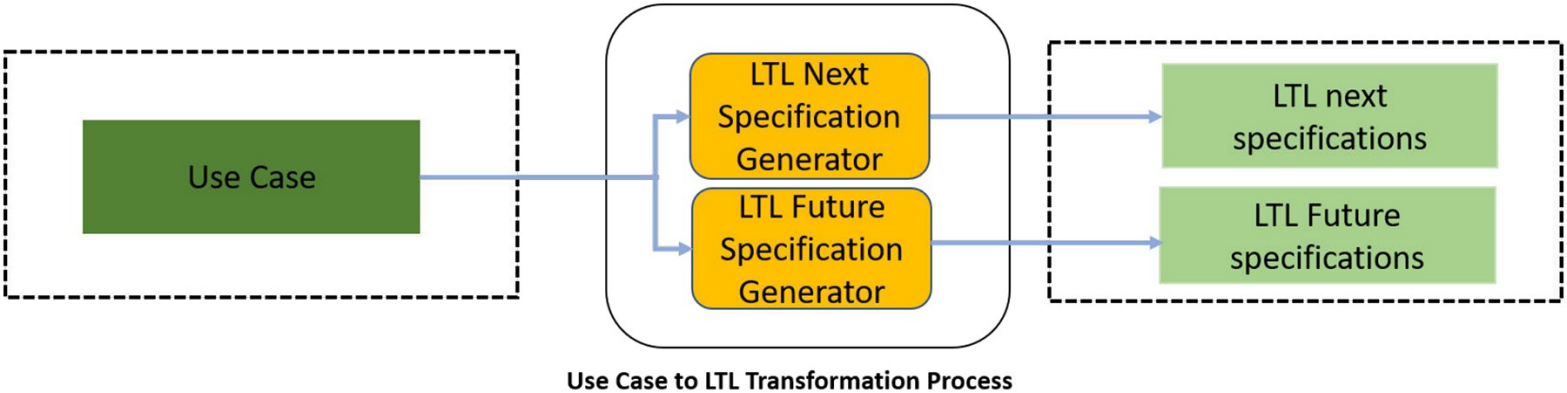
Use case to LTL transformation process.

The scenario lines of a use case description specify either an actor’s interaction with the software using an input symbol or system’s interaction with the actor using an output symbol. The input symbol is specified with the *input* identifier in the produced LTL formal specifications. Each of the specified *OutputSet* has a *label* and it is used to specify a particular output. The *OutputSymbol* in *OutputSet* holds the possible value for this output. In the start of a use case scenario, all of the *OutputSet* have a null value and this is marked as the *Initial*_*State* in the generated LTL formal specifications. The state is specified in the LTL formal specifications by *state*. LTL formal specifications are generated from a use case description by *LTLNextSpecificationGenerator* process and *LTLFutureSpecificationGenerator* process and these are described by Rule 1 and Rule 2 respectively. The block diagram of *Use Case to LTL Transformation* process is shown in Fig 7.

Rule 1 produces LTL formal specifications using the LTL next operator. It initializes the *OutputLabel* with all the specified *OutputSet* label’s value to *null*. It, then, scans all scenario lines one by one for the occurrence of actor, input symbol and output symbol. If any of these is found in the line being read then it enables the corresponding flag *isActor*, *isInput* and *isOutput* to *true*. It stores the read input symbol to *InputSymbol*_*read*_. The read output symbol is stored to *OutuputSymbol*_*read*_ and the value of corresponding label in *OuputLabel* is updated with the value of *OutuputSymbol*_*read*_. The value of *OutputLabel* is stored in *OutputLabel*_*beforeExtension*_ on reading the *Extension*_*Point* line and is reassigned to *OutputLabel* on reading the *End*_*Extension*_*Point* line. Whereas, the value of *OutputLabel* is stored in *OutputLabel*_*beforeAlternate*_ and it is reassigned to *OutputLabel* on reading the *Continue* line or *End*_*of*_*AlternateScenario* line. An LTL formula identified with an identifier *Formula*_*current*_ is defined by using the values of *OutputLabel*, *InputSymbol*_*read*_ and the value of *OutputSymbol*_*read*_. A keyword *LTLSPEC* is added in the start of *Formula*_*current*_ to make it appropriate for model checking using NuSMV model checker. The generated LTL formula identified as *Formula*_*current*_ is added to LTL formulas and is the final output of this process.

**Rule 1** LTL next specification generator process

 *isInput* ← *false*, *isActor* ← *false*, *isOutput* ← *false*

 String *OutputLabel*, *OutputLabel*_*beforeExtension*_, *OutputLabel*_*beforeAlternate*_,

 *InputSymbol*_*read*_, *OutputSymbol*_*read*_, *Formula*_*current*_

1: **for** *set* in *UC*.*OutputSet* **do**

2:  *set*.*OutputSymbols* ← *set*.*OutputSymbols* + null

3:  *OutputLabel* ← *OutputLabel* + *set*.*Label* + “= null”

4: **end for**

5: **for** *ℓ* in *UC*.*ScenarioLine* **do**

6:  **for** *inputsymbol* in *UC*.*InputSet* **then**

7:   **if** *ℓ* contains *inputsymbol* **then**

8:    *isInput* ← *true*

9:    *InputSymbol*_*read*_ ← *inputsymbol*

10:   **end if**

11:  **end for**

12:  **for** *set* in *UC*.*OutputSet* **do**

13:   **for** *outputsymbol* in *set* **do**

14:    **if** *ℓ* contains *outputSymbol* **then**

15:     *isOutput* ← *true*

16:     *OutputSymbol*_*read*_ ← *outputsymbol*

17:     **for** *set*_*available*_ in *UC*.*OutputSet* **do**

18:      **if** *set*.*Label* = *set*_*available*_ **then**

19:       Update *OutputLabel*.*set*.*Label* ← *OutputSymbol*_*read*_

20:      **end if**

21:     **end for**

22:    **end if**

23:   **end for**

24:  **end for**

25:  **for** *actor* in *UC*.*ActorSet* **do**

26:   **if** *ℓ* contains *actor* **do**

27:    *isActor* ← *true*

28:   **end if**

29:  **end for**

30:  **if** *ℓ*.typeof(*UC*.*ExtensionPointLine*) **then**

31:   *OutputLabel*_*beforeExtension*_ ← *OutputLabel*

32:  **end if**

33:  **if** *ℓ*.typeof(*UC*.*EndExtensionPointLine*) **then**

34:   *OutputLabel* ← *OutputLabel*_*beforeExtension*_

35:  **end if**

36:  **if** *ℓ*.typeof(*UC*.*AlternateScenarioLine*) **then**

37:   *isAlternate* ← *true*

38:   *OutputLabel*_*beforeAlternate*_ ← *OutputLabel*

39:  **end if**

40:  **if** *ℓ*.typeof(*UC*.*ContinueLine*) OR *ℓ*.typeof(“UC.EndAlternateScenarioLine”) **then**

41:   *isAlternate* ← *false*

42:   *OutputLabel* ← *OutputLabel*_*beforeAlternate*_

43:  **end if**

44:  **if** *isActor* AND *isInput* AND *isOutput* **then**

45:   **if** all *OutputSet*.*Label*.*value* = *null* **then**

46:    *Formula*_*current*_ ← “LTLSPEC G (state = Initial_State & input =” + *InputSymbol*_*read*_ + “− > X (” + *OutputSymbol*_*read*_ + “)”

47:   **else**

48:    *Formula*_*current*_ = “LTLSPEC G (” *OutputLabel* + “& input =” + *InputSymbol*_*read*_ + “−> X (” + *OutputSymbol*_*read*_ + “)”

49:   **end if**

50:  **end if**

51:  LTL formulas = LTL formulas + *Formula*_*current*_

52:  *isActor* ← *false*, *isOutput* ← *false*

53: **end for**

Rule 2 enlists the process to generate the LTL formulas using LTL future operator. It initializes the *Input*_*future*_ value to null in the start of the process. It scans all scenario lines one by one for the occurrence of actor, input symbol or output symbol. If any of these is read in the line being read then it enables the corresponding flag *isActor*, *isInput* and *isOutput* to *true*. It stores the read output symbol to *OutputSymbol*_*read*_. When an input symbol is read, the value of *Input*_*future*_ is assigned to *Input*_*beforefuture*_. The value of *Input*_*future*_ is then concatenated with ***X*** where ***X*** represents the LTL next operator and the read input symbol with a label *input* in the generated LTL formula.

**Rule 2** LTL future specification generator process

 boolean *isInput* ← *false*, *isActor* ← *false*, *isOutput* ← *false*

 String *Input*_*future*_, *Input*_*beforefuture*_, *Input*_*beforeExtension*_, *Input*_*beforeAlternate*_,

 *OutputSymbol*_*read*_, *Formula*_*current*_

 *Counter*_*input*_ ← 0, *isFirstWritten* ← *false*, *isAlternate* ← *false*

1: **for** *ℓ* in *UC*.*ScenarioLine* **do**

2:  **for** *inputsymbol* in *UC*.*InputSet* **do**

3:   **if** *ℓ* contains *inputsymbol*

4:    *isInput* ← *true*

5:    **if** *Counter*_*input*_ = 0 **then**

6:     *Counter*_*input*_++

7:     *Input*_*future*_ ← “(input =” + *inputsymbol* + “)”

8:    **else**

9:     *Input*_*beforefuture*_ ← *Input*_*future*_

10:     *Input*_*future*_ ← *Input*_*future*_ + “& X (input =” + *inputsymbol* + “)”

11:    **end if**

12:   **end if**

13:  **end for**

14:  **for** *set* in *UC*.*OutputSet* **do**

15:   **for** *outputsymbol* in *set* **do**

16:    **if** *ℓ* contains *outputSymbol* **then**

17:     *isOutput* ← *true*

18:     *OutputSymbol*_*read*_ ← *outputsymbol*

19:    **end if**

20:   **end for**

21:  **end for**

22:  **for** *actor* in *UC*.*ActorSet* **do**

23:   **if** *ℓ* contains *actor* **then**

24:    *isActor* ← *true*

25:   **end if**

26:  **end for**

27:  **if** *ℓ*.typeof(*UC*.*ExtensionPointLine*) **then**

28:   *Input*_*beforeExtension*_ ← *Input*_*future*_

29:  **end if**

30:  **if** *ℓ*.typeof(*UC*.*EndExtensionPointLine*) **then**

31:   *Input*_*future*_ ← *Input*_*beforeExtension*_

32:  **end if**

33:  **if** *ℓ*.typeof(*UC*.*AlternateScenarioLine*) **then**

34:   *isAlternate* ← *true*

35:   *Input*_*beforeAlternate*_ ← *Input*_*future*_

36:   *Input*_*future*_ ← *Input*_*beforefuture*_

37:  **end if**

38:  **if** *ℓ*.typeof(*UC*.*ContinueLine*) OR *ℓ*.typeof(*UC*.*EndAlternateScenarioLine*) **then**

39:   *isAlternate* ← *false*

40:   *Input*_*future*_ ← *Input*_*beforeAlternate*_

41:  **end if**

42:  **if** *isActor* AND *isInput* AND *isOutput* **then**

43:   *Formula*_*current*_ ← “LTLSPEC G (state = Initial_State &” + *Input*_*future*_ + “−> F (” + *OutputSymbol*_*read*_ + “)”

44:  **end if**

45:  LTL formulas ← LTL formulas + *Formula*_*current*_

46:  *isActor* ← *false*, *isOutput* ← *false*

47: **end for**

The value of *Input*_*future*_ is stored in *Input*_*beforeExtension*_ on reading the *UC*.*ExtensionPointLine* and is reassigned to *Input*_*future*_ when the *UC*.*EndExtenstionPointLine* is read. Whereas, on reading the *UC*.*AlternateScenarioLine*, the value of *Input*_*future*_ is stored in *Input*_*beforeAlternate*_ and the value of *Input*_*future*_ is updated with the value of *Input*_*beforefuture*_. While, on reading the *UC*.*ContinueLine* or *UC*.*EndAlternateScenarioLine* the value of *Input*_*future*_ is updated with the value of *Input*_*beforeAlternate*_. The LTL formula identified by the *Formula*_*current*_ identifier is produced by using the values of *state*, *Input*_*future*_ and *OutputSymbol*_*read*_. A keyword *LTLSPEC* is added in the start of *Formula*_*current*_ to make it appropriate for model checking using NuSMV model checker. The generated *Formula*_*current*_ is added to LTL formulas.

The time complexity of Rule 1 and Rule 2 is ***O***(*n*(*ip*+*os* (*op*)+*ac*)) where *n* denotes the number of scenario lines, *ip* denotes the number of input symbols, *os* denotes the number of *OutputSet*, *op* denotes the number of *output* symbols in an *os*. The variable *ac* denotes the number of actors in a use case description.

### Soundness of the proposed approach

The proposed approach consists of two transformation processes i.e. Use Case to Kripke Structure Transformation and Use Case to LTL Transformation. In the following paragraphs, we will discuss the soundness of these processes.

#### Soundness of use case to Kripke structure transformation process

This process produces a Kripke structure from the provided use case. The generated Kripke structure is well formed and deterministic in nature. Initially this process defines an initial state *s*_0_ and a dead state *d*_0_. These states are added to the states of the generated Kripke structure. All the generated states are labeled with unique bitvector of same length. All the input symbols are unique. The generated Kripke structure is deterministic. This process defines a unique initial state. There is only one transition defined for the read input symbol and the transitions for the remaining input symbols are defined and mapped to *d*_0_.

#### Soundness of use case to LTL transformation process

This process generates LTL formulas from given use case. A context free grammar has been defined using Extended Backus-Naur Form (EBNF) to verify the well formedness of the generated LTL formulas. The grammar is as follows:

*Ltlstart* = “*LTLSPEC*” *Ltlform* {“*LTLSPEC*” *Ltlform*}.
*Ltlform* = *Atomicprop* {*Binaryopr Atomicprop*}.
*Binaryopr* = “*U*” | “*R*” | “−>” | “&” | “|” | “=” | “!=”.
*Atomicprop* = “(”*Ltlform*“)” | *Unaryopr Ltlform* | “*TRUE*” | “*FALSE*” | *id*.
*Unaryopr* = “*X*” | “*G*” | “*F*” | “!”.
*id* = *alpha* {*alpha*}.
*alpha* = ‘*a*’‥‘*z*’ + ‘*A*’‥‘*Z*’ + ‘_’.

The generated LTL formulas for the examples have been parsed against this grammar and no error was found. Count of the generated LTL formulas is dependent on the number output symbols of given use case. Whereas, complexity of LTL formulas is dependent on number of input symbols, system action lines and *AlternateScenario* lines of the use case.

A process is called complete in terms of its ability to generate Kripke structure and LTL formulas from all use case constructs provided in the proposed template.

#### Completeness of use case to Kripke structure transformation process

The use case constructs include input symbols, output symbols, user action line, actor action line, AlternateScenario and EndAlternateScenario lines. The include and extend constructs are handled by Use Case Flattener process. This process replaces these to ExtensionPoint and EndExtenstionPoint lines. This process handles all use case constructs proposed in use case template. Rule 1 handles input symbols and output symbols of input use case. Rule 2 handles user action and system action lines. Rule 5 handles *AlternateScenario* line and Rule 6 handles Continue line. In addition to theses, Rule 7 handles *EndAlternateScenario* line. Whereas, *ExtensionPoint* and *EndExtensionPoint* lines are handled by Rule 8 and Rule 9 respectively.

#### Completeness of use case to LTL transformation process

This process consists of two rules and both rules handle all use case constructs. The use case constructs are treated in different contexts for the generation of LTL formulas by Rule 1 and Rule 2. Input symbols are handled by lines 6-11 of Rule 1 and lines 2-13 of Rule 2. Whereas, output symbols are handled by lines 1-4, 12-24 of Rule 1 and lines 14-21 of Rule2. *ExtensionPoint* and *EndExtensionPoint* lines are handled by lines 30-35 of Rule 1 and lines 27-32 of Rule 2. *AlternateScenario* line is handled by lines 36-39 of Rule 1 and lines 33-37 of Rule 2. *Continue* and *EndAlternateScenario* are handled by lines 40-43 of Rule 1 and lines 38-41 of Rule 2.

### Tool support

The proposed approach is implemented in the Use Case to Kripke Structure and LTL formulas generator tool (UCKSLTL) [28]. This tool takes a use case in the proposed template as an input and produces the resultant Kripke structure along with LTL specifications. The generated Kripke structure is presented in .*dot*, .*gml*, .*png* and .*smv* formats. The tool uses GraphViz API [29] to draw the generated Kripke structure. This tool verifies the syntactical structure of a use case against the proposed template. These features simplifies the user task to document a use case description and the transformation process.

## Example

**Fig 8:**
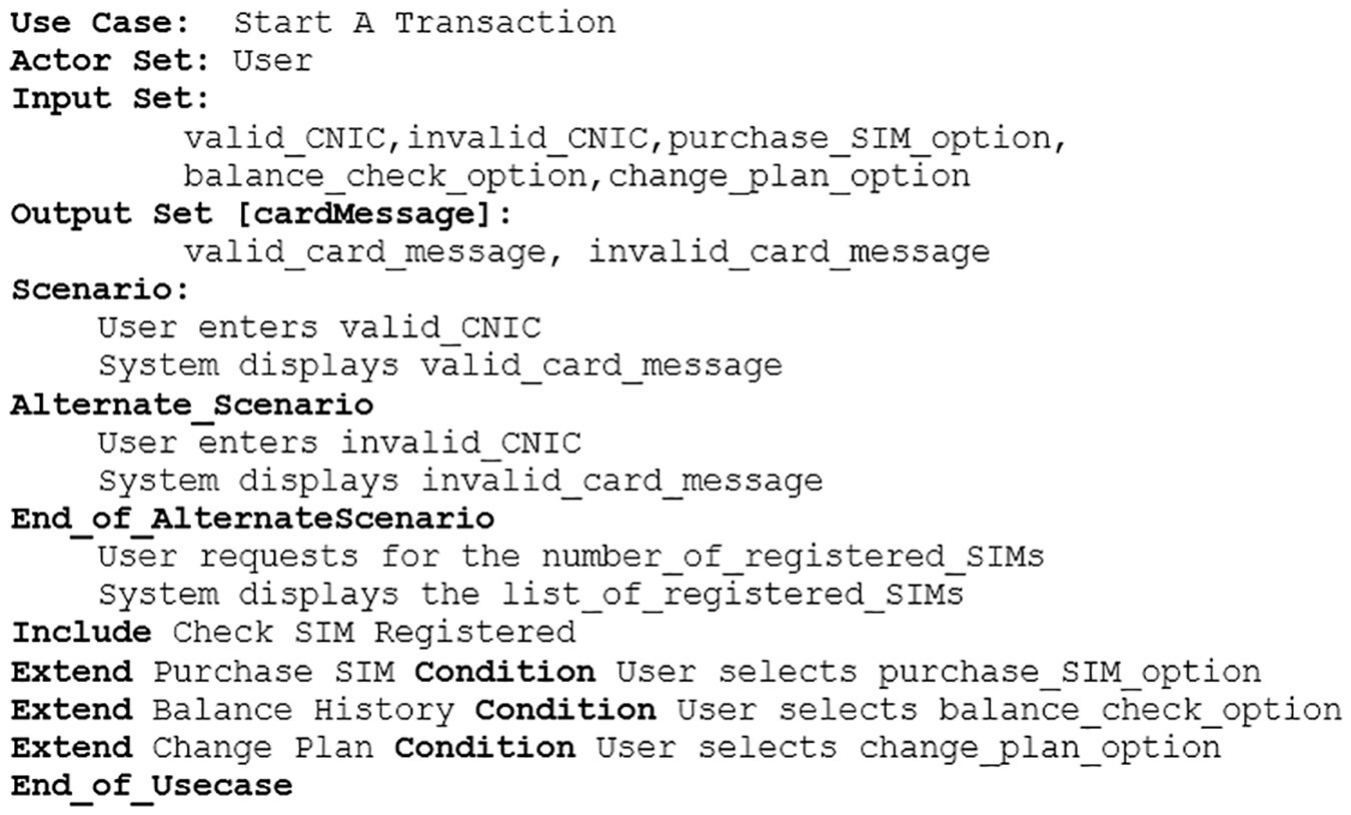
A SIM vending machine start a transaction use case description.

**Fig 9:**
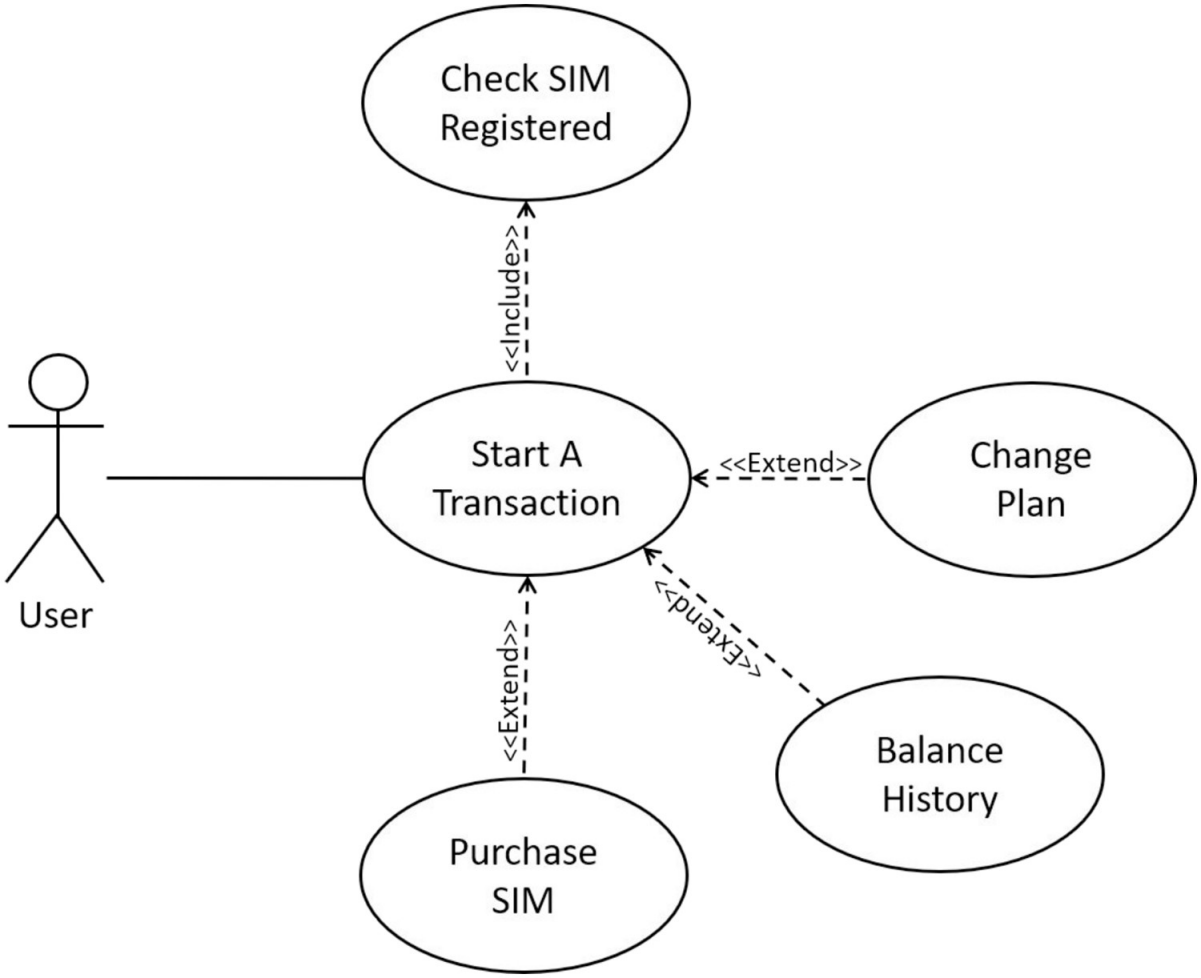
A SIM vending machine start a transaction use case diagram.

The proposed approach has been used to transform use case descriptions of a number of examples which can be retrieved from UCKSLTL weblink [28]. We select Subscriber Identification Module (SIM) vending machine example to show the working of proposed approach in this paper. A SIM vending machine, works as a kiosk. It facilitates its user to check for a registered SIM, purchase a new SIM, view balance history and update call plan after adding a Computerized National Identification Card (CNIC) number. The use case description for SIM vending machine is shown in Fig 8. The use case diagram for this is shown in Fig 9.

**Fig 10:**
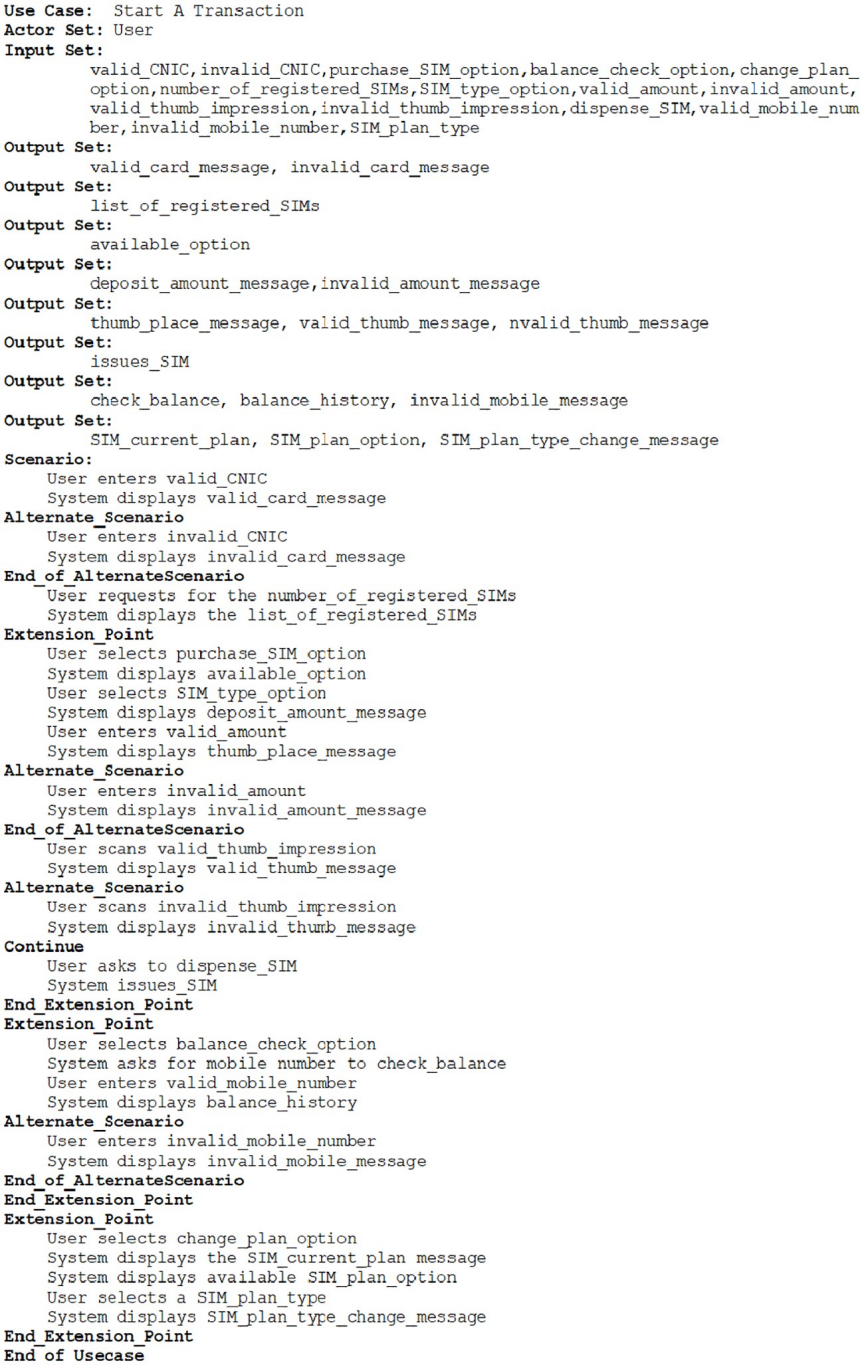
A SIM vending machine start a transaction use case detailed description.

The proposed approach requires to add use case description of the included and extended use cases descriptions in the use case description of the use case including or extending them. This addition is also performed by the tool. However, the use case description after addition of the use case descriptions being included and extended is shown in Fig 10 to provide the reader an insight into this artifact.

**Fig 11:**
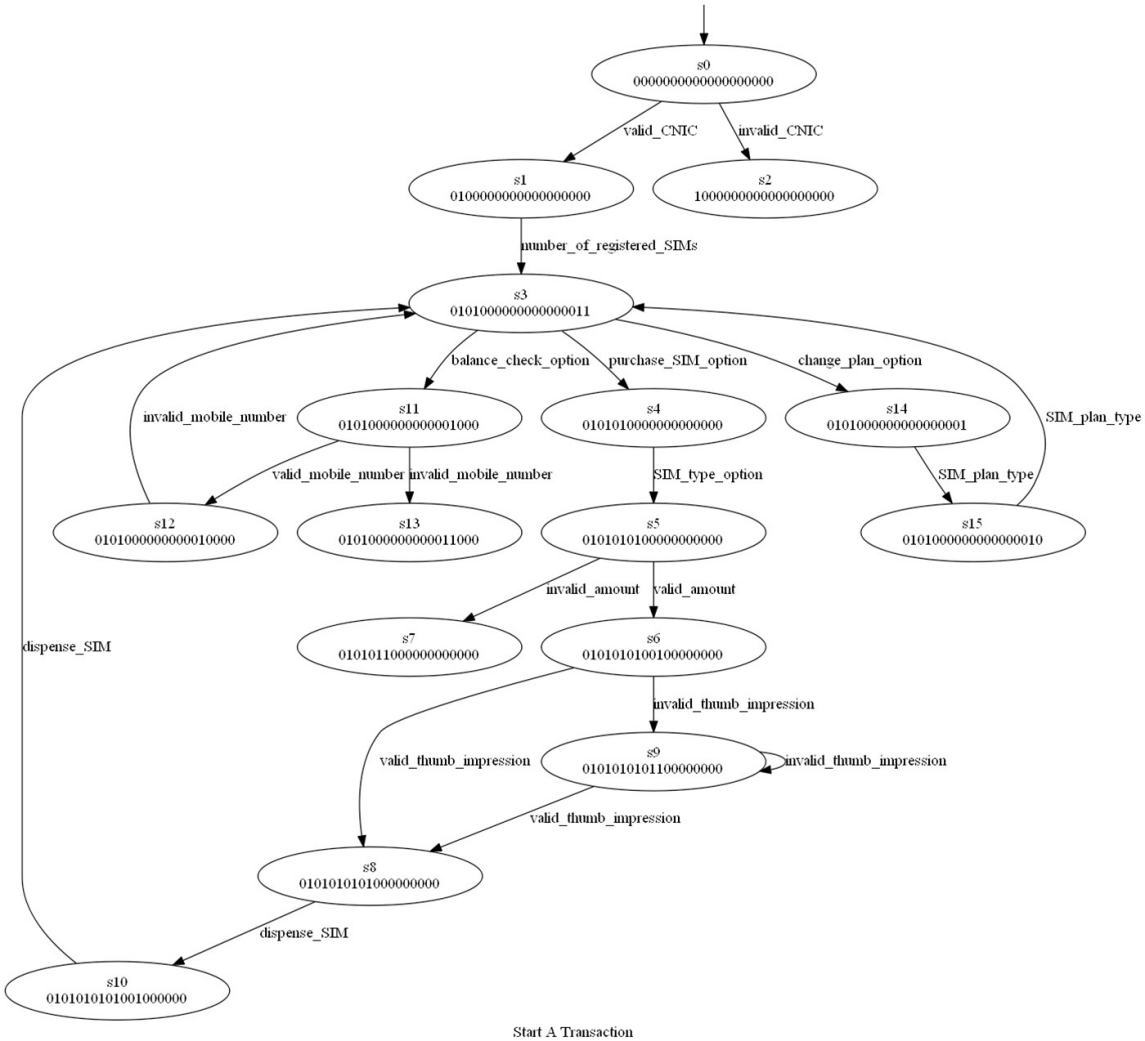
A SIM vending machine start a transaction Kripke structure.

The corresponding Kripke structure instance is shown in Fig 11. The exceptional behavior of system being developed is mapped to a dead state. In the generated Kripke structure, the dead state and the transitions mapping to it are not shown to make this figure readable.

A set of LTL formal specifications is generated by this approach for SIM vending machine example. Some of the generated LTL formulas are listed here:

- *LTLSPEC* ***G***(*state* = *Initial_State* & *input* = *valid_CNIC* → ***X***(*cardMessage* = *valid_card_message*))

The software displays a valid card message to the user if the user provides a valid CNIC to the software at the initial state.

- *LTLSPEC* ***G***(*cardMessage* = *valid_card_message* & *checkSIMMessage* = *null* & *purchaseSIMOptionMessage* = *null* & *amountMessage* = *null* & *thumbMessage* = *null* & *issueSIMMessage* = *null* & *balanceHistoryMessage* = null & *changePlanMessage* = *null* & *input* = *number_of_registered_SIMs* → ***X***(*checkSIMMessage* = *list_of_registered_SIMs*))

The software displays the list of registered SIMs to the user if the user asks the software to provide the number of registered SIM after the provision of valid CNIC to the software.

- *LTLSPEC* ***G***(*state* = *Initial_State* & (*input* = *valid_CNIC*) & ***X*** (*input* = *number_of_registered_SIMs*) → ***F*** (*checkSIMMessage* = *list_of_registered_SIMs*))

A user will get the list of registered SIMs from the software by providing a valid CNIC and asking the software to provide number of registered SIM as input.

- *LTLSPEC* ***G*** (*state* = *Initial_State* & (*input* = *valid_CNIC*) & ***X*** (*input* = *number_of_registered_SIMs*) & ***X*** (*input* = *change_plan_option*) → ***F*** (*changePlanMessage* = *SIM_current_plan*))

A user will get the information of SIM current plan after providing a valid CNIC, number of registered SIM and SIM plan as input to the software.

The approach presented in this paper transforms a use case description into corresponding Kripke structure and LTL formal specifications. Two different approaches are used in this study: one is to generate a Kripke structure from a use case description and the other one produces LTL formal specifications from the same use case description. Both of the generated formalism correspond to the same software and can be used by a model checker like NuSMV as an input for the validation purposes. For the validation purpose the generated Kripke structure and the LTL formal specifications were provided to the NuSMV tool. Upon execution NuSMV did not generate any counterexample. This validates the generated kripke structure and LTL formal specifications.

This approach is domain independent and requires software requirements artifact, in the proposed template, for the transformation process. Whereas the other available approaches require additional artifacts like domain model, sequence diagram, interaction diagram, activity diagram or business rules definition along with the software requirements artifact for the transformation process. The user of this approach also does not require the skills to specify the software requirements artifact in some specialized specification language.

## Scalability of the proposed approach

**Fig 12:**
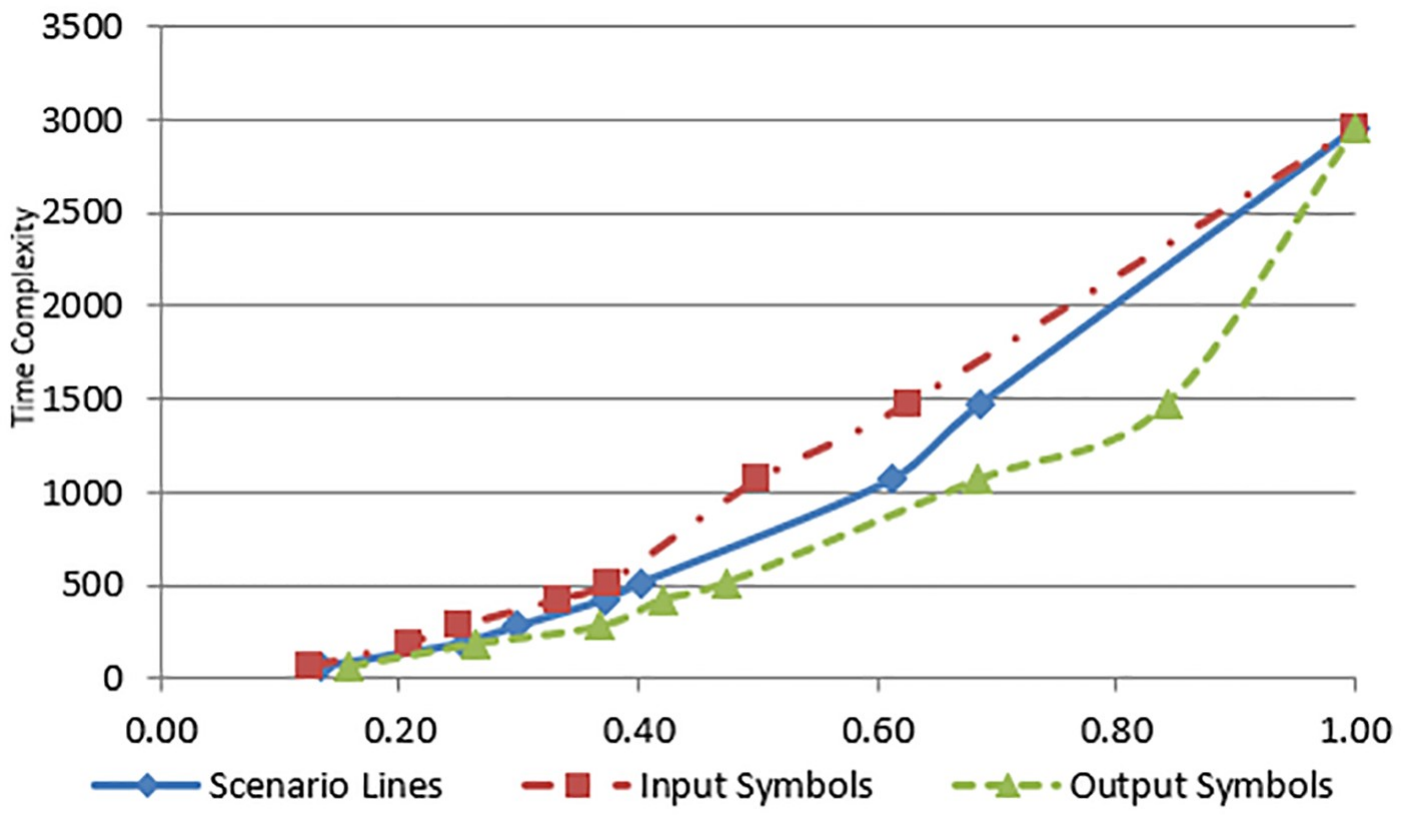
Time complexity to use case parameters.

**Fig 13:**
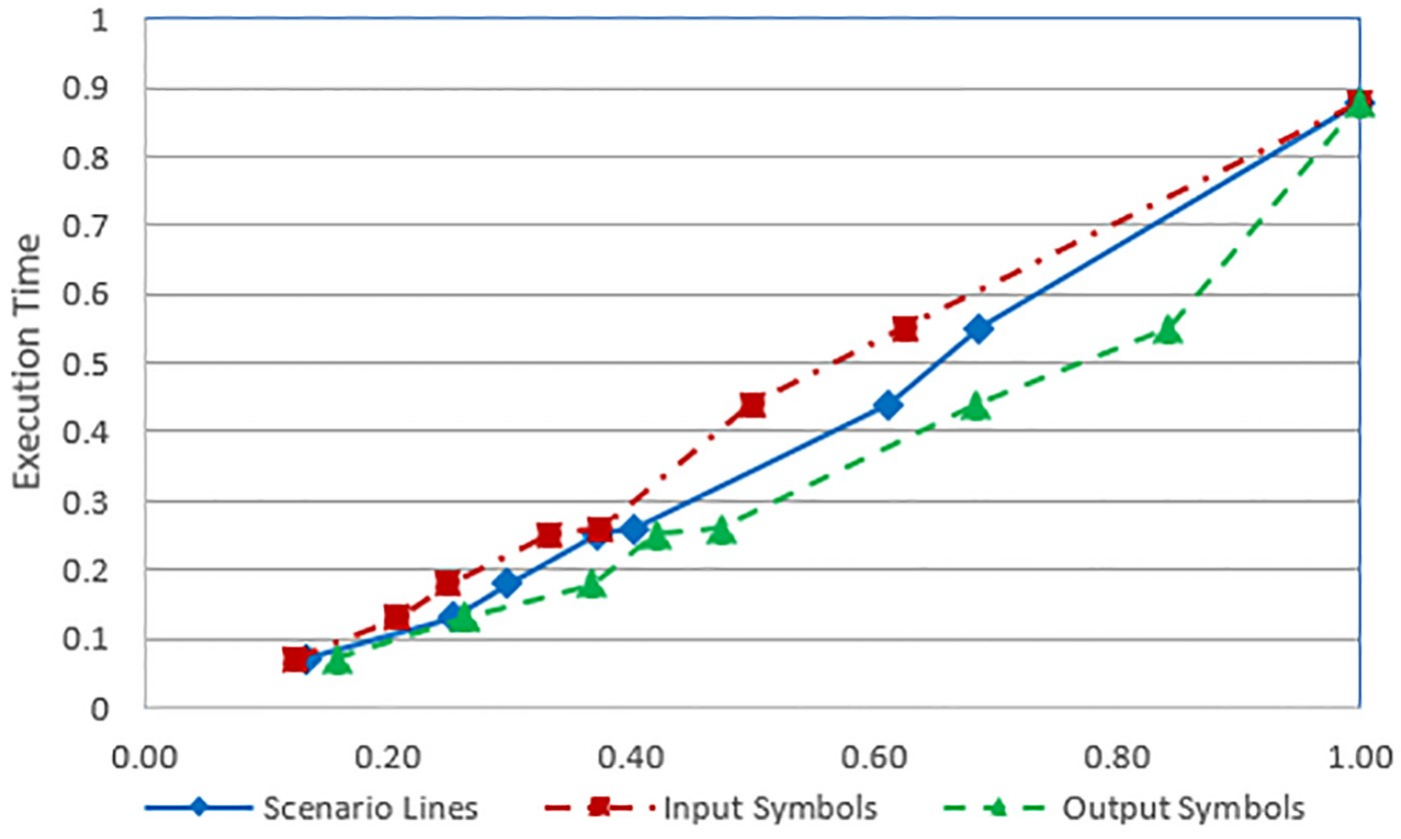
Execution time to use case parameters.

**Fig 14:**
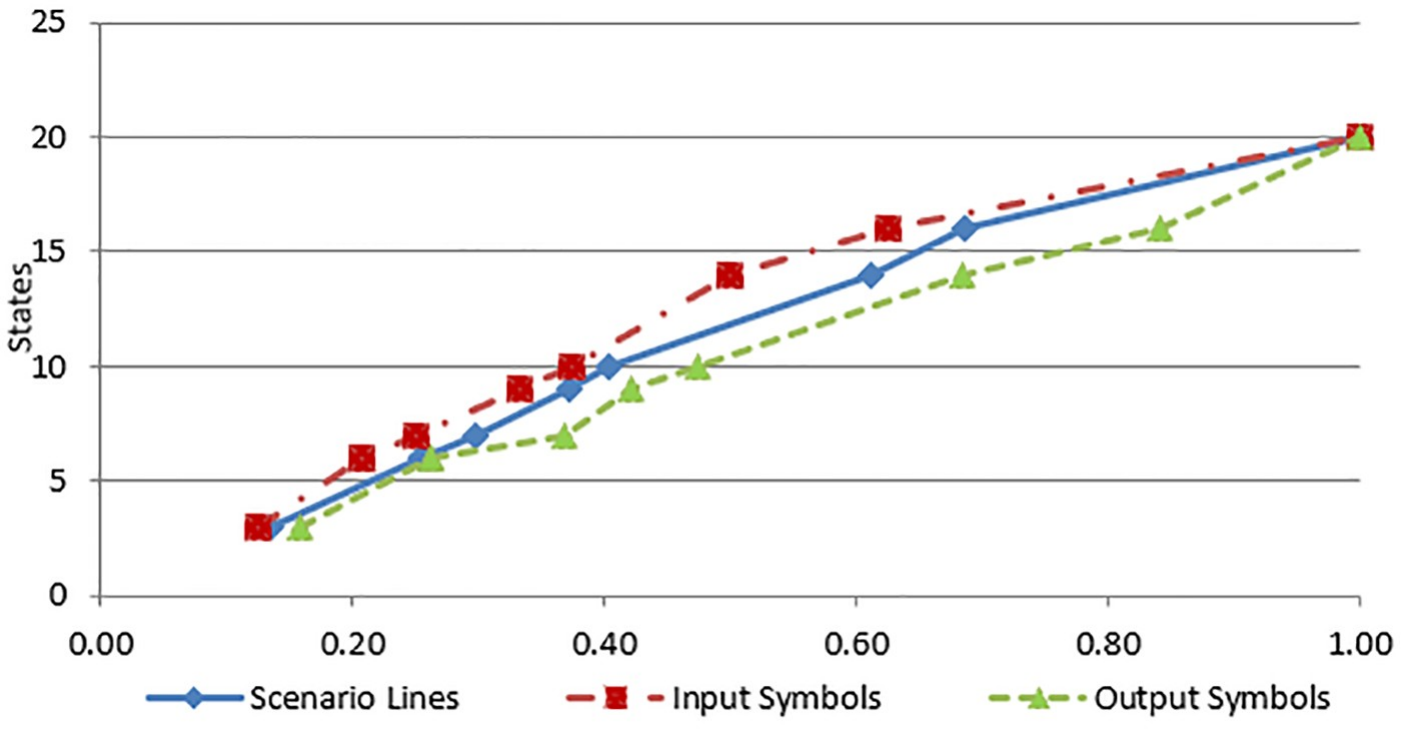
Kripke structure states to use case parameters.

**Fig 15:**
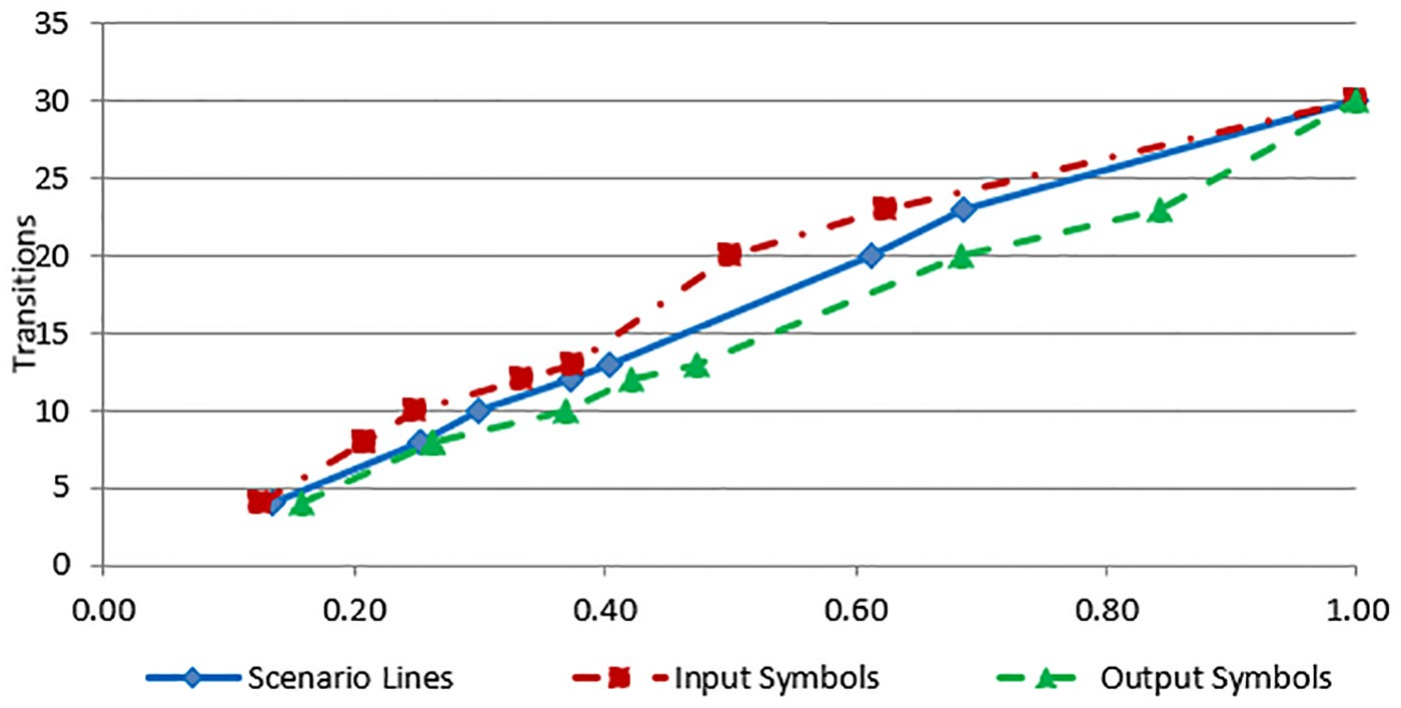
Kripke structure transitions to use case parameters.

In this section, we present some preliminary results to assess the scalability of the proposed approach for larger case studies. The ATM cash withdrawn example, the SIM vending machine example and two other case studies have been used for scalability analysis of the proposed approach. The time complexity and the execution time of the proposed transformation process depend on the following four parameters of the input use case: (a) number of actors (b) number of input symbols (c) number output symbols and (d) number of scenario lines. However, in our case studies, there is only one actor in each use case. The experiments have been performed on an Intel Core2 Duo P8600 machine with 8 GB RAM, running 64-bit Microsoft Windows 7 Professional operating system. The transformation process for the example and each case study have been executed 500 times and a mean execution time has been computed to eliminate slight variance due to operating system processes and threads scheduled at a specific time. Table 1 lists the execution times against the selected input parameters, as well as the number of states and transitions of the generated Kripke structure for each case study. Fig 12 shows a growth in time required (as given by time complexity formula) against the use case description parameters. The values of use case description parameters have been normalized in the range 0 to 1. The values of use case parameters are on the x-axis and the values of time complexity are on the y-axis of the graph. Likewise Fig 13 shows the relationship between use case parameters and the actual execution time. The values of use case parameters are on the x-axis and the values of execution times on the y-axis of the graph. The graph shown in Fig 12 reflects an increase in the time complexity values as the values of use case parameters increase. It can be seen from both figures that the growth in time requirement is not linear, it is increasing slightly more rapidly. This observation is consistent with the time complexity formula given in *Use Case to Kripke Structure Transformation Process* sub-section which shows that the worst-case time complexity is quadratic. Another aspect of scalability relates to the size of generated Kripke structure. The last two columns of Table 1 show the numbers of states and transitions of the generated Kripke structure. Figs 14 and 15 show growth in the number of states and transitions, respectively, against the use case parameters. The values of use case parameters have been normalized before plotting these graphs. Both graphs show linear growth in the size of generated Kripke structure against the size of input use case.

**Table 1:** Use case parameters and Kripke structure.

Name		Input Symbols	Output Symbols	Scenario Lines	Time Complexity	Execution Time (ms)	States (Q)	Transitions (T)
**ATM cash withdrawn**		6	7	21	283	0.19	8	50
**SIM Vending Machine**		15	16	46	1472	0.55	16	23
**Customer Work Flow**	**Story 1**	6	7	20	280	0.18	7	10
	**Story 2**	9	9	27	513	0.26	10	13
	**Story 3**	8	8	25	425	0.25	9	12
	**Story 4**	5	5	17	187	0.13	6	8
	**Story 5**	5	5	17	187	0.13	6	8
**Touch’D**	**Make Profile**	24	19	67	2948	0.88	20	30
	**View Contact**	12	13	41	1066	0.44	14	20
	**Call A Contact**	3	3	9	63	0.07	3	4

## Related work

In this section, we review the approaches that transform use case description into corresponding formal notations.

Somé et al. [4] propose to generate a state transition graph from a use case description and a domain model of a software. The domain model evolves with the maturity of the software development process. In addition, the generated transition graph does not consider the outputs of the software. This reduces the use of this generated transition graph for basic level of verification activities. Moreover, this also requires the additional efforts to reformulate a transition graph with the evolution of the domain model.

Kalnins et al. [5] generate a multi-layered model from a use case description. The use case description in the case of Kalnins et al. is required to be expressed in Requirements Specification Language (RSL) [30]. This use case description along with the UML activity and interaction diagrams are used to produce an architectural model. The generated architectural model includes application logic, business logic and data access layers. These layers are populated with UML component, interface, dependency, class and package instances. This approach is also supported by a tool. Though Kalnins et al. make a significant contribution but Smialek et al. [31] comprehend the usage of RSL with manual annotation of noun, verb, subject and predicate in a use case description. Their approach generates a sequence diagram from this annotated software requirements expressed using RSL. This approach requires manual efforts for the labeling process. Furthermore, the obtained precision is not scalable for supporting large scale industrial projects. Software validation plays an equally important role that is why it needs to be considered duly. Unfortunately, the approach presented by Smialek et al. [31] does not consider this aspect. Whereas, Scandurra et al. [6] proceed by prioritizing the validation aspect. The authors used RUCM framework for achieving this objective and eventually Scandurra et al. provided corresponding abstract state machine. The same framework of RUCM has also been used by another group of researchers Yue et al. [9] but the difference is that Yue et al. apply restricted rules and UCMeta for constructing sentences needed for use case description. The achievement of the authors primarily revolves around the transformation of use case description into the target domain and activity diagrams. Though the authors along with the utility of aToucan [32] contributed in simplifying the whole setup. However, this approach requires comparatively higher degree of expertise for documenting the use case description using RUCM. It seems impractical for a common user to practice this approach which discourages its integration into related areas of research.

Zaman et al. [19] transform a use case description into a Kripke structure. This approach requires the specification of use case description in a proposed template. This template requires the calculation of corresponding binary values for the output symbols of the software. Moreover, this approach does not handle the use case relationships.

Singh et al. [33] propose a technique that requires the creation of UML class and sequence diagrams prior to the formal transformation. After forming of these two artifacts they are able to formalize the static and dynamic views of a software in Z language. A software static view is extracted from a use case description with the support of a class diagram. Whereas, the software dynamic view is generated from the sequence diagrams. UML class and sequence diagrams are built during the design stage of the software development process. A correction at this stage of the development process is much expensive and also requires reformation of other software development artifacts.

A use case can describe only a single functionality offered in a software. Whereas, a software constitutes a number of functionalities. The above discussed approaches do not consider the software level constraints. Software level constraints can be defined with the help of Object Constraint Language (OCL) [34] and is used by Chu et al. [10] for formal transformation. They have used USL to document a use case description. The pre- and post-conditions of use case description are required to be expressed in OCL. This use case description along with UML class diagram is used to build a Labeled Transition System (LTS) [35] by using defined domain meta concepts and utility functions. This approach is expensive in terms of writing pre- and post-conditions of a use case in OCL, specifying use case description in terms of domain meta concepts and analytical skills to build a class diagram. The generated labeled transition system seems not to be aligned with the formal system due to absence of initial and final states in its formal setup that requires an explicit initial state to start its computation.

The approaches discussed so far focus on architectural arrangements of a software and ignore the software’s business environment as well its constraints. This aspect has been considered by researchers and a number of approaches have been proposed to formalize informal requirements. Business environment of a software influence the design and working of a software. These can be represented by using business rules and domain ontology and are used by the following approaches for the formal transformation of informal software requirements.

Selway et al. [36], use General Architecture for Text Engineering (GATE) to process business rules expressed in a controlled natural language to generate the preliminary Semantics of Business Vocabulary and Business Rules (SBVR) model [37] with the assistance of a domain expert. The limitation of this model lies in its continuous and unconditional reliance over the domain expert and its limited vocabulary.

Li et al. [3] further investigated process of informal to formal requirements transformation but with the slight difference of using the Web Ontology Language (OWL) in the transformation process. This approach is limited in impact due to its nature of being domain specific. Consequently, it requires consistent additional efforts for the description of object, rules and relations.

The aforementioned discussed approaches transform informal specifications to formal specifications. Most of these approaches depend on the usage of a restricted natural language like RSL or RUCM, prior to the transformation process. The disadvantage of limited vocabulary and restricted rules compromise inherent features of simplicity and ease of use. Other approaches require the understanding and expertise in constraint languages like OCL are expensive as these constraints are defined on artifacts other than the requirements document. A number of approaches require the formation of artifacts like domain, sequence, interaction and activity diagrams. These diagrams are built later in the design stage of the software development process. Any correction at this stage is expensive and require the reformation of these artifacts prior to the re-transformation process. The approaches to transform informal requirements in the light of software business environment are domain specific and are based on the definition of business objects and their relations. Such domain specific approaches require the services of a domain expert for the realistic definition of business environment.

Considering the identified limitations including understanding of specialized languages, requirement of additional skill set, formation of other software development artifacts and definition of software environment, there is a need for an approach that allows to specify the software requirements in natural language, using requirements stage artifacts and is domain independent.

A comparison of the proposed approach with the existing approaches based on required input, generated output, required additional artifacts and additional skills to practice the approach is analyzed. This analysis is provided in Table 2.

**Table 2:** Analysis of existing approaches with the proposed approach.

Proposed by	Input	Output	Limitations
Somé et al.	Use Case Description, Domain model	State transition graph	Domain model of a system evolves with the evolution of the system. The generated state transition graph does not consider the output symbols of the software.
Smialik et al.	Use Case Description	Sequence diagram	The user of this approach requires the specification skills using RSL language. In addition, the generated sequence diagram is semi-formal UML artifact and cannot be used for model checking directly.
Kalnins et al.	Use Case Description, Activity and Interaction diagrams	Architectural model	The generated architectural diagram is semi-formal artifact and it cannot be used for the model checking purposes. In addition, the required activity and interaction diagrams along with a use case description are design phase artifacts. The correction cost at the design phase is relatively high than the correction cost at the requirements analysis phase. Moreover, the user of this approach requires the specification skills using RSL language.
Scandurra et al.	Use Case Description	Abstract state machine	The user of this approach requires the specification skills using RUCM framework. Moreover, the generated abstract machine requires to add more details of the system to make it suitable for the verification and validation purposes.
Zaman et al.	Use Case Description	Kripke structure	This approach requires to calculate the corresponding binary values for the output symbols. In addition, it does not require use case relationships.
Yue et al.	Use Case Description	Domain model and Activity diagram	The user of this approach requires the specification skills using UCMeta. In addition, the generated domain and activity diagram are semi-formal UML artifacts and cannot be used for the model checking.
Singh et al.	Use Case Description, Class diagram and Sequence diagram	Z specification	The additional artifacts including class and sequence diagram are design phase artifacts. The correction cost at the design phase is relatively high than the correction cost at the requirements analysis phase. In addition, the generated formalism is a requirements phase artifact.
Chu et al.	Use Case Description, Class diagram	Labeled Transition System	Additional skills are required to specify use case specification using USL and employing use case contracts using OCL. Moreover, a labeled transition may have infinite number of states and there are not initial and final states in a labelled transition system. The initial and final states are required for the formal verification activities.
Selway et al.	Business rules	SBVR model	Specification skills are required to specify business rules using GATE. In addition, the services of a domain expert are required.
Proposed Approach	Use Case Description	Kripke structure and LTL formulas	This approach does not handle the nested alternate scenarios.

It can be observed from Table 2 that most of these approaches require additional artifacts like domain model, sequence diagram, activity diagram and interaction diagram along with use case description prior to the transformation process. These diagrams are created at the design stage of the software development process. It can also be seen that some of these approaches require use case description specification in some specialized specification language like USL, RUCM or RSL. This aspect adds additional requirement for a user of these approaches. A number of these approaches are domain specific and require continuous support of domain expert for domain concepts definition. In comparison to these approaches, the proposed approach requires the software artifact itself specified in the proposed template using natural language. This template is simple enough and requires from the user to identify software inputs and outputs, those are defined at requirements elicitation stage.

The objective of this study is to generate a Kripke structure and LTL formal specifications. For this purpose, only those attributes of a use case description are considered which are useful for the target formalism and these attributes are common in other existing use case templates. In proposed approach, additional attributes of use case description like pre-/post- condition, trigger, etc. are not considered as they do not contribute in the transformation process.

## Conclusion

In this paper, we proposed an approach that transforms informal software requirements, specified as use cases, to corresponding formal requirements, i.e., LTL formal specification and a Kripke structure. The proposed approach handles use case relationships including *include* and *extend* which allows the proposed approach to transform a use case model instead of a single use case description. Moreover, this approach performs transformation at meta-model level. The user of this approach does not require any additional skills like understanding of constraint language, e.g., OCL or specialized natural language specification languages like RUCM or USL. The approach does not require any additional artifacts like domain model, sequence diagram, activity diagram, interaction diagram or business rules definition. An example of SIM vending machine is used to demonstrate this approach. The generated formal specifications, i.e., LTL formal specifications and a Kripke structure are validated using the NuSMV model checker which produces no counterexamples.

In future, the presented approach can be extended to make it compatible with the other existing use case templates.
